# Analyses of genome architecture and gene expression reveal novel candidate virulence factors in the secretome of *Phytophthora infestans*

**DOI:** 10.1186/1471-2164-11-637

**Published:** 2010-11-16

**Authors:** Sylvain Raffaele, Joe Win, Liliana M Cano, Sophien Kamoun

**Affiliations:** 1 The Sainsbury Laboratory, John Innes Centre, Norwich NR4 7UH, UK

## Abstract

**Background:**

*Phytophthora infestans *is the most devastating pathogen of potato and a model organism for the oomycetes. It exhibits high evolutionary potential and rapidly adapts to host plants. The *P. infestans *genome experienced a repeat-driven expansion relative to the genomes of *Phytophthora sojae *and *Phytophthora ramorum *and shows a discontinuous distribution of gene density. Effector genes, such as members of the RXLR and Crinkler (CRN) families, localize to expanded, repeat-rich and gene-sparse regions of the genome. This distinct genomic environment is thought to contribute to genome plasticity and host adaptation.

**Results:**

We used *in silico *approaches to predict and describe the repertoire of *P. infestans *secreted proteins (the secretome). We defined the "plastic secretome" as a subset of the genome that (i) encodes predicted secreted proteins, (ii) is excluded from genome segments orthologous to the *P. sojae *and *P. ramorum *genomes and (iii) is encoded by genes residing in gene sparse regions of *P. infestans *genome. Although including only ~3% *of P. infestans *genes, the plastic secretome contains ~62% of known effector genes and shows >2 fold enrichment in genes induced *in planta*. We highlight 19 plastic secretome genes induced *in planta *but distinct from previously described effectors. This list includes a trypsin-like serine protease, secreted oxidoreductases, small cysteine-rich proteins and repeat containing proteins that we propose to be novel candidate virulence factors.

**Conclusions:**

This work revealed a remarkably diverse plastic secretome. It illustrates the value of combining genome architecture with comparative genomics to identify novel candidate virulence factors from pathogen genomes.

## Background

*Phytophthora infestans*, the causal agent of the potato and tomato late blight disease, is a successful cosmopolitan plant pathogen. Ever since the Irish potato famine in the middle of the nineteenth century, *P. infestans *has been recognized as one of the most problematic plant pathogens with a global impact on both commercial and subsistence agriculture [1]. This oomycete pathogen is recalcitrant to low input disease management and requires costly chemical treatments to be managed [2]. Part of *P. infestans *success is accounted for by its biological lifestyle and remarkable capacity to rapidly adapt to overcome resistant plants [3]. On infected plants, it continuously produces a large number of asexual spores, including sessile aerially dispersed sporangia and motile zoospores, resulting in polycyclic infections and fast spreading late blight epidemics [4]. In addition, in many regions of the world, *P. infestans *reproduces sexually resulting in increased genetic diversity and extended survival in the field [2]. Based on these biological and epidemiological features, McDonald and Linde concluded that *P. infestans *is a plant pathogen with a high evolutionary potential that can rapidly evolve virulence on resistant plants [3].

Similar to a wide range of animal and plant pathogens, *P. infestans *secretes proteins, termed effectors, that facilitate parasitic colonization by altering host plant physiology and suppressing immunity [5-7]. *P. infestans *effector proteins target different sites in host plant tissue [5,6,8]. First, some effectors act in the extracellular space where they interfere with apoplastic plant defenses. Inhibitors of plant extracellular proteases and glucanases are such apoplastic effectors [9-13]. Other effectors, such as small cysteine-rich proteins (SCRs), are also thought to function in the apoplast but their effector activities remain mostly unknown [5,14]. Second, a large number of *P. infestans *effectors, classified as cytoplasmic effectors, are delivered inside host cells using *N*-terminal secretion and host-translocation signals [5,6,15]. This is the case for members of the RXLR and Crinkler (CRN) families. A subset of the RXLR effectors is recognized inside plant cells by intracellular immune receptors of the nucleotide-binding leucine-rich repeat (NB-LRR) family (so-called resistance or R proteins), resulting in the induction of hypersensitive cell death and immunity [16,17].

Evolutionary and comparative genomics analyses revealed that *Phytophthora *effector genes have undergone accelerated patterns of birth and death evolution with evidence of extensive gene duplication and gene loss in the genomes of *P. infestans*, *P. sojae*, and *P. ramorum *[15,18-20]. For instance, in *P. infestans*, only 16 out of the 563 predicted RXLR genes are part of the "core ortholog" gene set (genes residing in 1:1:1 orthologous genome segments between *P. infestans*, *P. sojae*, and *P. ramorum*) [15]. Also, effector genes frequently show signatures of positive selection with extensive non-synonymous sequence substitutions, leading to high rates of amino acid polymorphisms [19,21,22]. In *P. infestans*, the RXLR and CRN gene families are among the most expanded relative to *P. sojae *and *P. ramorum *[15]. These RXLR and CRN genes mostly populate expanded regions of the *P. infestans *genome that have low gene density and a high abundance of repeats in marked contrast to the housekeeping "core ortholog" gene set that occupy gene-dense and repeat-poor regions [15]. Haas *et al *proposed that these gene-poor repeat-rich loci are dynamic regions of the genome that underpin the evolutionary potential of *P. infestans *by promoting genome plasticity and enhancing genetic variation of effector genes. Similarly, virulence genes occur in plastic repeat-rich and telomeric regions in various pathogens, which is thought to increase genetic and epigenetic variation and could result in accelerated evolution [23-25].

All known oomycete effectors carry *N*-terminal signal peptides for secretion outside pathogen cells [5,6,8]. Although signal peptide sequences are highly degenerate, robust computational prediction algorithms enable a systematic survey of the secreted protein catalog (the secretome) from the genome sequence of a given organism [26]. In particular, the SignalP program that was developed using machine learning methods [27], can assign signal peptide prediction scores and cleavage sites to unknown amino acid sequences with a high degree of accuracy [28,29]. This program turned out to be particularly useful for the prediction of effectors from *P. infestans *and other filamentous pathogens as numerous SignalP predictions have been validated experimentally [30-35]. A combination of computational prediction methods was used recently to generate a database of the secretome from 158 fungal and oomycete organisms [36].

In the *P. infestans *genome, a majority of core ortholog genes occur in gene dense regions (GDRs) and are excluded from gene sparse regions (GSRs), which are in contrast enriched in effector genes [15]. This distinctive genome organization offers a unique opportunity to identify novel candidate virulence genes. Furthermore, although the secretome of *P. infestans *includes several hundred candidate effectors belonging to multiple classes, additional families of secreted proteins have not been characterized in much detail [15]. In this study, we used a computational approach to catalog the secretome of *P. infestans *strain T30-4. We then defined and identified the "plastic secretome" as the set of secreted protein genes that (i) do not reside in segments orthologous to *P. sojae *and *P. ramorum *genomes, and (ii) reside in the repeat-rich GSRs. This pipeline resulted in 561 proteins (~3% of the total proteome), of which 398 have already been annotated as effectors by Haas *et al. *[15]. Because the pipeline identified many *in planta*-induced genes and ~62% of all previously predicted *P. infestans *effectors, we concluded that the remaining 163 proteins from the "plastic secretome" are enriched in novel candidate effectors. In particular, we highlight 19 genes that are induced *in planta *and distinct from known effector families. These analyses implicate trypsin-like serine proteases, berberine-bridge enzymes, carbonic anhydrases, small cysteine-rich proteins and repeat-containing proteins as novel candidate virulence factors.

## Results

### Prediction and annotation of *Phytophthora infestans *secretome

To identify the secretome of *P. infestans *(set of proteins predicted to be soluble secreted), we predicted signal peptides using the well-validated SignalP v2.0 and v3.0 programs and sub-cellular targeting using TargetP and PSORT (see methods). To ensure stringent standards, only proteins predicted secreted by the four methods were considered further. To remove proteins likely to be retained into *P. infestans *plasma membrane we excluded those for which a transmembrane domain was predicted after the signal peptide cleavage site by TMHMM (see methods). In total, 1,415 of the 18,155 proteins of *P. infestans *were predicted to form the secretome (Additional file 1). To complement existing annotation, we performed detection of protein domains using Pfam and Superfamily 1.73 HMM model databases and automated GeneOntology (GO) terms mapping using Blast2GO server (Additional file 1).

### Major functional categories enriched in *Phytophthora infestans *secretome

**Figure 1:**
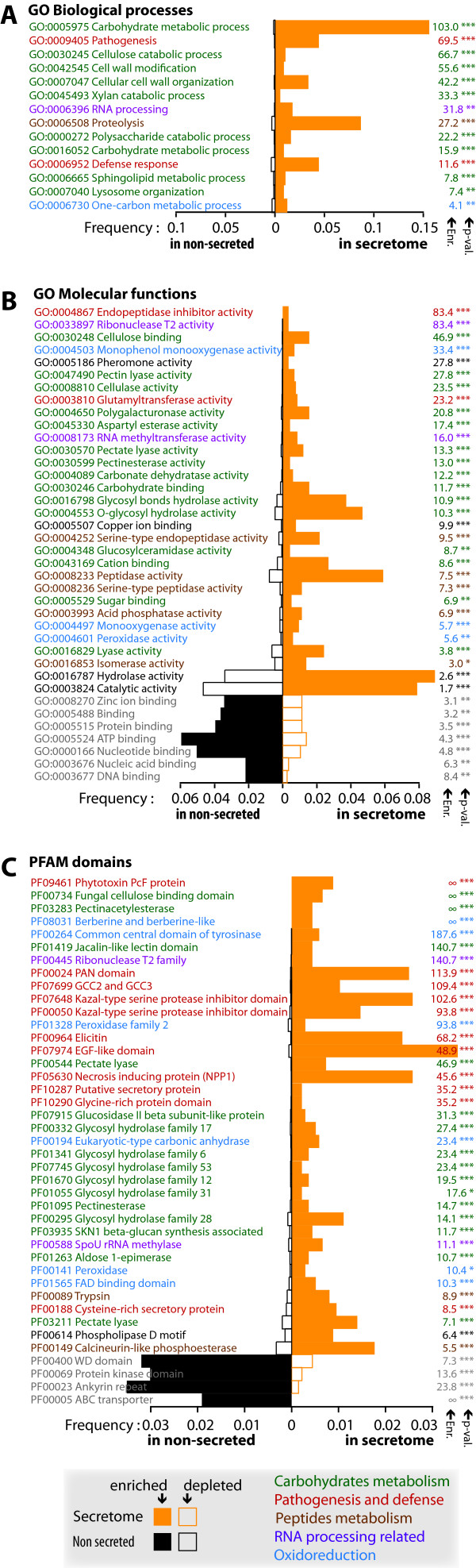
**Gene Ontologies and Pfam domains enriched in the *Phytophthora infestans *secretome**. The graphs show the number of proteins annotated with GO biological process (**A**), GO molecular function (**B**) and Pfam domains (**C**) and their frequency (number of proteins with annotation/total number of proteins) in the *P. infestans *secretome (yellow bars) and non-secreted proteins (black bars). Only GO and Pfam domains significantly enriched or depleted in the secretome are shown (chi-square test with Bonferroni correction, p-value -p-val- indicated on the leftmost part of the panels: ***, p-value < 0.01; **, p-value < 0.05; *, p-value > 0.1). GO and Pfam domains were classified by decreasing enrichment in the secretome (Enr., see methods). Full bars indicate ontology or domain enriched in the secretome, empty bars indicate ontologies or domain depleted from secretome. Ontologies and domains were color-coded for easier reference. Enr., enrichment or depletion fold; p-val, p-value of chi-square test.

To document biological functions enriched in the *P. infestans *secretome, we compared the frequency of occurrence of Pfam domains and GO terms in the secretome to the rest of the proteome using chi-square tests (see methods). We found 15 "Biological process" ontologies, 31 "Molecular function" ontologies and 43 Pfam domains to be enriched in the *P. infestans *secretome. Seven "Molecular function" ontologies and 4 Pfam domains were depleted from secretome (Figure 1).

Carbohydrate metabolic processes (GO:0005975, also GO:0016052) showed the highest enrichment among biological processes in the *P. infestans *secretome compared to the rest of the proteome (Figure 1A, green). Related biological processes enriched in the secretome include cell wall modification (GO:0042545) and organization (GO:0007047) processes, as well as catabolism of polysaccharides (GO:0000272), specifically cellulose (GO:0030245) and xylan (GO:0045493). In addition, most of the proteins associated with the sphingolipid metabolic process (GO:0006665) and lysosome organization (GO:0007040) ontologies show sequence similarity to glycosyl hydrolases indicating that these two ontologies are also mostly related to carbohydrate metabolism in *P. infestans *secretome. Consistently, 15 "molecular function" ontologies directly or indirectly related to sugar metabolism are enriched in the secretome (Figure 1B, green). Sugar binding (GO:0030248, GO:0030246, GO:0005529) and sugar modification activities (GO:0047490, GO:0008810, GO:0004650, GO:0030570, GO:0030599, GO:0004089, GO:0016798, GO:004553) are indeed predominantly found in the *P. infestans *secretome. Furthermore, a majority of proteins associated to glucosylceramidase activity (GO:0004348), and cation binding (GO:0043169) ontologies show similarity to glycosyl hydrolases. Most of the proteins associated to aspartyl esterase activity (GO:0045330) and lyase activity (GO:0016829) show similarity to polygalacturonases and polysaccharide lyases respectively. This enrichment indicates that sensing extracellular sugar and degrading host cell wall are major functions of the *Phytophthora *secretome as illustrated by several previous studies [37-39]. Finally, 15 Pfam domains enriched in the secretome correspond to enzymes predicted to act on sugars (Figure 1C, green), either as monomers (PF01419 on mannose) or polysaccharides, including cellulose (PF00734, PF01341), α- and ß-1,3 glucans (PF01055, PF00332), ß-1,4 glucans (PF07745), xyloglucans (PF01670), rhamnoglucans (PF00295) and pectin (PF03283, PF00544, PF01095, PF03211). Aldose 1-epimerase (PF01263), responsible for interconversion of D-glucose and other aldoses, completes the list of carbohydrate metabolism-related domains enriched in the *P. infestans *secretome.

Pathogenesis (GO:0009405) and defense response (GO:0006952) are biological process ontologies highly enriched in *P. infestans *secretome (Figure 1A, red). The corresponding proteins include some with similarity to elicitins. The molecular function ontology with the highest enrichment in the secretome, endopeptidase inhibitor (GO:0004867), corresponds to Kazal-like serine protease inhibitors, which have been linked to the infection process as apoplastic effectors [10,11,40] (Figure 1B, red). Proteins corresponding to the glutamyltransferase activity (GO:0003810) show similarity to transglutaminase elicitor-like proteins harboring the Pep-13 pathogen associated molecular pattern [41]. The Kazal-type serine protease inhibitor domain is also found among Pfam domains enriched the in secretome (PF07648, PF00050) (Figure 1C, red), together with elicitin domain (PF00964) and necrosis inducing protein domain (PF05630). The Pfam domain showing the highest enrichment in the secretome is the cysteine-rich PcF domain (PF09461) that forms a two-alpha helices domain rich in acidic residues and was reported to cause leaf necrosis [42]. The PAN domain (PF00024) is another cysteine-rich domain enriched in the *P. infestans *secretome. The PAN domain occurs in the Cellulose-Binding Elicitor-Like protein of *Phytophthora parasitica *that causes necrosis and activates immunity in plants [43]. Several other Pfam domains enriched in the *P. infestans *secretome are cysteine-rich domains of unclear functions, such as the GCC domain (PF07699), EGF-like domain (PF07974) and the domains of unknown function PF00188 and PF10287. Secreted proteins containing these cysteine-rich domains could play a role in plant infection similar to known small cysteine-rich proteins [14]. Generally, the secretome appears enriched in small (50 to 150 amino acids) proteins and in proteins rich in cysteine (>5%) (Additional file 2). Similarly, the *P. infestans *secretome shows higher frequency of proteins with elevated (>10 or >30%) glycine content (Additional file 2). One such example is the IPIB family [44] and its corresponding Pfam domain PF10290 (Figure 1C).

Proteolysis (GO:0006508) is a biological process ontology enriched in the *P. infestans *secretome (Figure 1A, brown). Consistently, serine type peptidase activity (GO:0004252, GO:0008236) and peptidase activity (GO:0008233) are molecular function ontologies that are also enriched in the *P. infestans *secretome (Figure 1B, brown). Acid phosphatase activity (GO:0003993) regroups another type of hydrolases enriched in the *P. infestans *secretome. Pfam domains implicated in peptide hydrolysis, namely trypsin domain (PF0089) and calcineurin domain (PF00149), which show similarity to acid phosphatases, are enriched in the secretome (Figure 1C, brown). In addition, proteins associated to isomerase activity ontology (GO:0016853) mainly show similarity to peptidyl-prolyl cis-trans isomerase or disulfide isomerases. These enzymes are known to accelerate energetically unfavorable cis/trans isomerization of the peptide bond preceding a proline to catalyze protein folding [45,46].

Surprisingly, RNA processing (GO:0006396) appears as a biological process enriched in the *P. infestans *secretome (Figure 1A, purple). Consistently, ribonuclease T2 (GO:0033897) and RNA methyltransferase activity (GO:0008173) are molecular function ontologies enriched in the secretome (Figure 1B, purple). The ribonuclease T2 (PF00445) and SpoU rRNA methylase (PF00588) are Pfam domains also enriched in the secretome (Figure 1C, purple). RNA cleavage by ribonuclease T2 was shown to be implicated in defense and self-incompatibility processes [47]. Some of these proteins might be effectors that are translocated inside plant cells to alter host transcription or DNA/RNA metabolism. Extracellular nucleases have been described in the fungi *Ustilago maydis *and *Aspergillus *spp. [26,48].

Proteins related to oxidoreduction were also particularly abundant in the *P. infestans *secretome. Secreted proteins classified under the one-carbon metabolic process ontology (GO:0006730) (Figure 1A, blue) show similarity to carbonic anhydrase enzymes, catalyzing the conversion of carbon dioxide and water to bicarbonate and protons. The corresponding Pfam domain (Eukaryotic-type carbonic anhydrase, PF00194) is enriched in the *P. infestans *secretome (Figure 1C, blue). Monooxygenase activity (GO:0004497) and monophenol monooxygenase activity (GO:0004503) are molecular function ontologies enriched in the secretome (Figure 1B, blue). Also enriched in the secretome are tyrosinase Pfam domain (PF00264), found in copper monooxygenases involved in the formation of pigments and polyphenolic compounds, and peroxidase Pfam domains (PF00141, PF01328). FAD-binding domain (PF01565) and berberine-like domain (PF08031), which occur in the same set of secreted proteins, complete the list of oxidoreduction-related domains enriched in the secretome.

Other ontologies enriched in the *P. infestans *secretome include generic activities such as catalytic (GO:0003824) and hydrolase (GO:0016787) activities, associated largely to predicted glycosyl hydrolases. Copper ion binding (GO:0005507) is another molecular function enriched in the secretome. The pheromone activity (GO:0005186) enriched in the secretome is found in proteins similar to temptins, which mediates protein-cell surface contact during fertilization in mollusks [49]. A Phospholipase D (PLD) motif (PF00614) is among the Pfam domains enriched in the *P. infestans *secretome. *Phytophthora *PLD activities were proposed to be involved in zoospore encystment [50] and host membrane modification [51] but these secreted PLDs could target host membranes.

Molecular function ontologies depleted from the *P. infestans *secretome (Figure 1B, grey) are generic binding activity (GO:0005488) and more specifically zinc ion binding (GO:0008270), protein binding (GO:0005515) and nucleotide- and nucleoside-binding (GO:0003677, GO:0003676, GO:0000166, GO:0005524). Protein-protein interaction Pfam domains such as WD (PF00400) and ankyrin repeat (PF00023) are depleted from the *P. infestans *secretome, together with the protein kinase domain (PF00069) and ABC transporter domain (PF00005).

### Delimitation of gene dense and gene sparse regions in the *P. infestans *genome

**Figure 2:**
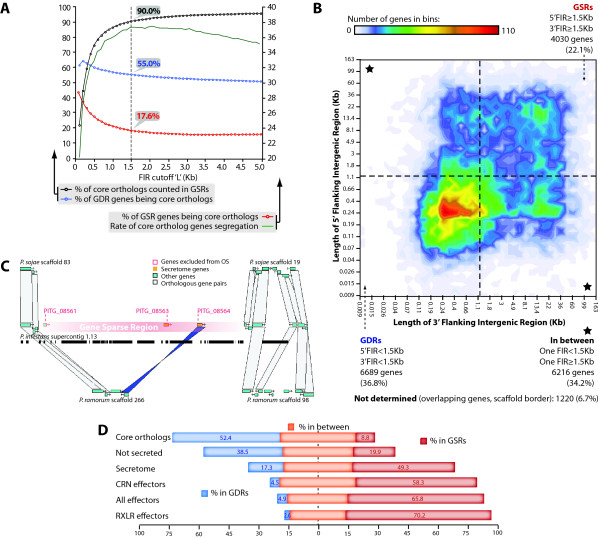
**Delimitation and effector content of *Phytophthora infestans *gene sparse regions (GSRs)**. **A) **Simulation of core ortholog gene segregation. Genes with both flanking intergenic regions (FIRs) longer than a value 'L' were considered as gene-sparse region (GSR) genes, whereas genes with both FIRs below L were considered as gene-dense region (GDR) genes. To quantitatively define GSRs, the % core orthologs among total genes falling in GDRs (blue) and GSRs (red) was calculated for values of L ranging from 100 bp to 5 kb. Core ortholog segregation rate was defined as the difference between the core ortholog content of the GDRs vs. GSRs (green). The percentage of core orthologs assigned to GDRs is shown as a black line. The highest core ortholog genes segregation rate was obtained for L = 1.5 kb. **B) **Distribution of *P. infestans *genes according to the length of their FIRs. All *P. infestans *predicted genes were sorted into 2-variable bins according to their 3'FIR (Y-axis) and 5'FIR (X-axis). The number of genes in bins is shown as a contour graph with a color code. The 1.5 kb limit for GSRs genes (dotted lines) delimits three groups of genes: genes in GDRs, GSRs, and in between (corresponding genes features and numbers are indicated in labels). **C) **A sample window from the *P. infestans *genome browser illustrating typical examples of GDRs and GSR (red background). In this 80 kb region, core ortholog genes are exclusively found in GDRs, secretome genes (yellow) and genes excluded from orthologous segments (OS, red box) are excluded from GDRs. **D) **Distribution of gene groups into the GDRs and GSRs of *P. infestans*. The proportion of non-secreted, secretome, known effectors, RXLR effector genes and CRN effector genes that occur in GSRs (red, with % indicated), GDRs (blue with % indicated) and in between (yellow) is shown.

Because the GSRs of the *P. infestans *genome are highly enriched in RXLR and CRN effector genes, we hypothesized that this property could be used to identify novel effector candidates. First, we needed to determine quantitative parameters that distinguish between GDRs and GSRs. To achieve this, we simulated core ortholog genes content in GDRs and GSRs (as % of total genes falling in each of these regions) using values of the length 'L' of flanking intergenic regions (FIRs) between genes ranging from 100 bp to 5 Kb (Figure 2A, blue and red lines respectively). Genes with both FIRs above L were considered GSR genes, whereas genes with both FIRs below L were considered GDR genes. Core ortholog segregation rate was defined as the difference between the core ortholog content of the GDRs vs. GSRs (green line). For low L values, many core orthologs were excluded from the GDRs since only very tightly packed genes were assigned to them. On the other hand, with larger L values, more genes were assigned to GSRs progressively reducing the proportion represented by the core orthologs. The highest segregation value was obtained for L = 1.5 kb. At this cutoff, 90% of the core orthologs were assigned to GDRs (black line) and constituted 55% of the GDR genes. In contrast, at L = 1.5 kb, only 17.6% of GSR genes were core orthologs. We therefore selected L = 1.5 kb for subsequent analyses because this value provided the best segregation between the core ortholog and effector genes into the GDR and GSR genomic compartments.

The 1.5 kb cutoff delimits four coherent gene pools when combined with the 2-variables binning representation previously performed by Haas *et al. *[15] (Figure 2B). The GDRs (genes with 5'FIR and 3'FIR < 1.5 kb) contain 6689 genes representing 36.8% of *P. infestans *genes. The GSRs (genes with 5'FIR and 3'FIR > 1.5 kb) include 4030 genes, corresponding to 22.1% of the genes. The other two quadrants group genes with asymmetric FIRs, one shorter than 1.5 kb and the other one longer. We counted 6216 (34.2% of the genome) genes residing at the border of GDRs and GSRs. Finally, 1220 genes (6.7% of the genome) were omitted because they lack one resolved FIR (locate at one border of scaffolds) or overlap with other genes.

An example of a genome browser view further illustrates the organization of a representative genome region into GDRs and GSRs (Figure 2C). This 80 kb area of *P. infestans *supercontig 1.13 contains a 60 kb GSR flanked by short GDRs. As opposed to GSR genes, all the GDR genes belong to genome segments orthologous to the *P. sojae *or *P. ramorum *genomes. All the secreted protein genes in this region occur in the GSR.

### Gene sparse regions are enriched in secreted proteins

GSRs contain 49.3% of the secretome genes even though they contain only 22.1% of the total *P. infestans *genes (Figure 2D). Consistent with previous analyses by Haas et al. [15], GSRs contain 65.8% of the effector genes, and more specifically 70.2% of the RXLR and 58.3% of the CRN genes. Compared to the whole genome, the GSRs show a two-fold enrichment in secreted protein genes, and a three-fold enrichment in effector genes.

In addition, 82.8% of secretome, 95.1% of effector, 97.4% of RXLR and 95.5% of CRN genes are excluded from the GDRs (occur in both the GSRs and at GDR/GSR borders). Of the known effectors, only 4.9% are found in the GDRs, with only 14 out of 540 RXLR effector genes and 6 out of 132 CRN genes.

### The "plastic secretome" of *P. infestans*: secretome genes excluded from genome segments orthologous to *P. sojae *or *P. ramorum *and residing in GSRs

**Figure 3:**
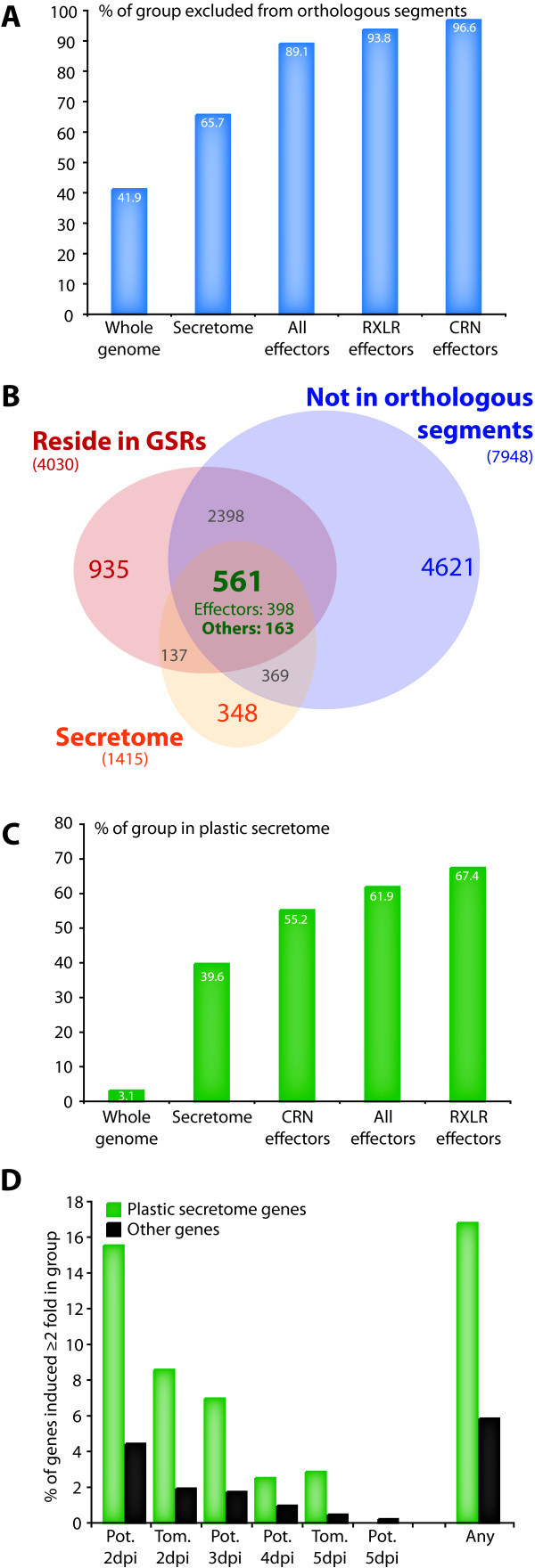
**Characterization of the *Phytophthora infestans *"plastic secretome"**. **A) **Frequency of *P. infestans *genes excluded from orthologous segments between *P. infestans *and either the *P. sojae *or *P. ramorum *genome. The proportion (% of gene group) of all, secretome, known effectors, RXLR effector and CRN effector genes is shown. **B) **Venn diagram illustrating the number of *P. infestans *genes (i) residing in GSRs and (ii) not in genome segments orthologous between the three *Phytophthora *species and (iii) belonging to the secretome. This set of three criteria defines the plastic secretome. The *P. infestans *plastic secretome consists of 561 genes: 398 known effector genes and 163 others. **C) **Percentage of various *P. infestans *gene groups found in the plastic secretome (as a % of the whole gene group). **D) **The plastic secretome is enriched in *in planta*-induced genes. The proportion of either plastic secretome (green) or non-plastic secretome (grey) genes induced *in planta *is shown. Genes induced at any of the time points tested are also shown ('Any'). Tom., infected tomato; Pot., infected Potato; dpi, days post-inoculation.

One defining feature of *P. infestans *effector genes is that they have significantly diverged from their counterparts in *P. sojae *and *P. ramorum *and are typically excluded from orthologous segments [15,19]. Orthologous segments (OS) are defined as genome segments derived from a common ancestor without large rearrangements, therefore containing genes showing homology, collinearity, conserved order and orientation in different species [52,53] (Additional file 3). We found that although only 41.9% (7948) of the total genes and 65.7% of the secretome genes are excluded from segments orthologous between at least two of the examined *Phytophthora *species, this proportion reaches 89.1%, 93.8% and 96.6% for all effector, RXLR and CRN genes, respectively (Figure 3A). We therefore hypothesized that we could significantly enrich in candidate effector genes using the combination of three criteria: (i) secreted protein, (ii) exclusion from OS, and (iii) occurrence in the GSRs or FIR not determined. In total, 561 genes fulfilled these three criteria (Figure 3B). Genome regions showing frequent re-arrangements, particularly in pathogenic bacteria, have been referred to as "plasticity zones" [54,55]. We therefore refer to the 561 gene set identified here as the "plastic secretome" of *P. infestans *to reflect their localization in plastic genome regions.

### The plastic secretome is highly enriched in effectors

Of the 561 genes assigned to the plastic secretome, 398 (70.9%) are annotated as effectors. Also, even though the 561 genes correspond to less than 3.1% of the whole genome, they include 61.9% of all known effector genes (67.4% of RXLR genes and 55.2% of CRN genes, Figure 3C and additional file 4). This clearly indicates that the plastic secretome is highly enriched in effectors and that the remainder 163 genes are likely to be enriched in novel candidate virulence genes.

### Genes from the plastic secretome are enriched in genes induced *in planta*

To identify candidate virulence genes among the genes from the plastic secretome, we used the whole-genome microarray expression data of *P. infestans *infection time course on potato and tomato [15]. Overall, the genes from the plastic secretome showed a higher proportion of genes induced *in planta *relative to the remainder of the genes (Figure 3D). In particular, during the early biotrophic phase of infection (2 dpi of potato or tomato) 8-16% of the genes from the plastic secretome are induced relative to less than 4.5% of the remaining genes (Figure 3D). In total, 95 of the 561 genes from the plastic secretome were classified as induced in at least one of the *in planta *time points tested (Additional files 1 and 5).

### *In planta *induced genes from the plastic secretome underpin novel candidate virulence genes

We examined in more details 19 genes from the plastic secretome that have not been previously annotated as effector genes and are induced *in planta *(Table 1, Additional file 6). Five candidates were annotated as cell wall degrading enzymes (CWDEs): PITG_02545 and PITG_08563 show similarity to pectin lyases, PITG_20953 has an aldose 1-epimerase domain found in some groups of glycoside hydrolases, PITG_22758 is related to arabinofuranosidase, and PITG_22899 has a Jacalin-like lectin domain predicted to bind mannose. Four candidates have other predicted enzymatic activity, including trypsin-like serine protease activity (PITG_02700), oxidoreductase activity (PITG_02930 berberine-bridge enzyme and PITG_18284 carbonic anhydrase) and putative mannose processing activity (PITG_22638). Two candidates are effector-like proteins: PITG_23138 is a truncated RXLR effector that was missed in earlier annotations [15] and PITG_16958 possess the Pep13 motif found in transglutaminase elicitors. Three candidates are repeat-containing proteins (RCPs): PITG_06957 and PITG_17477 have glycine-rich motifs while PITG_06212 harbors lysine-rich repeats. Two candidates are small cysteine-rich proteins (SCRs, PITG_04202, PITG_07213) not previously described. Finally, three candidates (PITG_01659, PITG_07586, PITG_21363) do not have significant similarities to known proteins and sequence motifs. Some of these candidates are described in more details hereafter.

**Table 1:** Main features of the 19 novel candidate virulence genes from *P. infestans *plastic secretome.

Gene	**FIRs (Kb)**^**a**^	**Swissprot BlastP (e-value)**^**b**^	**Pfam (e-value)**^**c**^	**IF 2 dpi**^**d**^	**Comments**^**e**^
**PITG_01659**	2248 - 24885	No hit	No hit	2.0x	
**PITG_02545**	1657 - 14983	Pectinesterase (7e^-63^)	PF01095 Pectinesterase(1e^-48^)	5.7x	CWDE
**PITG_02700**	13752 - 14329	Chymotrypsinogen B2 (5e^-22^)	PF00089 Trypsin (1e^-41^)	3.5x	Detailed in Figure 4
**PITG_02930**	6988 - 2141	6-hydroxy-D-nicotine oxidase (2e^-13^)	PF01565 FAD binding (2e^-20^)PF08031 BBE (9e^-08^)	6.5x	Detailed in Figure 5
**PITG_04202**	8616 - 13236	No hit	No hit	9.8x	Detailed in Figure 7 SCR (94aa, 6.4% C)
**PITG_06212**	26186 - 23867	No hit	No hit	3.5x	Detailed in Figure 8 RCP (232 aa, 11.2% G)
**PITG_06957**	24029 - 3888	No hit	No hit	2.1x	Detailed in Figure 8 RCP (247aa, 21.5% G)
**PITG_07213**	3344 - 18922	HEAT repeat-containing protein 1 homolog (3e^-04^)	No hit	2.6x	Secreted SCR (114 aa, 6.1% C)
**PITG_07586**	14222 - 25733	No hit	PB012569 (8e^-07^)	2.6x	
**PITG_08563**	29269 - 8440	Probable pectin lyase F-2 (4e^-56^)	PB000314 (4.e^-20^)PF00544 Pectate lyase C (6e^-12^)	2.0x	CWDE
**PITG_16958**	6840 - 15261	No hit	PB013434 Pfam-B_13434 (7e^-154^)	9.8x	Pep13 motif of transglutaminase elicitor
**PITG_17477**	ND - 5531	No hit	No hit	2.0x	Detailed in Figure 8 RCP (374aa, 36.1% G)
**PITG_18284**	35643 - ND	Carbonic anhydrase (2e^-21^)	PF00194 Carbonic anhydrase (7e^-30^)	5.3x	Detailed in Figure 6
**PITG_20953**	7474 - 9088	Putative glucose-6-P 1-epimerase (8e^-29^)	PF01263 Aldose epimerase (5e^-22^)	2.0x	CWDE
**PITG_21363**	33627 - ND	No hit	No hit	3.5x	
**PITG_22638**	ND - ND	Mannose-P-dolichol utilization defect 1 (2e^-11^)	PF04193 PQ-loop (3e^-13^)	2.0x	
**PITG_22758**	108426 - 12121	Alpha-N-arabino-furanosidase B (4e^-102^)	PF09206 Alpha-L-arabino-furanosidase B (7e^-127^)	2.8x	CWDE
**PITG_22899**	2414 - 21611	No hit	PF01419 Jacalin (3e^-09^)	4.6x	CWDE
**PITG_23138**	32957 - 16047	No hit	No hit	2.5x	Truncated RXLR effector

^a^Length of flanking intergenic regions: ND, not determined. ^b ^Best BlastP hit against swissprot database. ^c^Pfam domains found. ^d^Gene induction fold (IF) 2 days post inoculation (dpi) in potato: expressed as fold of gene expression in mycelia. ^e^aa, amino-acid, % of cysteine or glycine residues indicated in some cases, CWDE, Cell Wall Degrading Enzyme, SCR, RCP

### Secreted trypsin-like serine proteases related to glucanase inhibitor proteins

**Figure 4:**
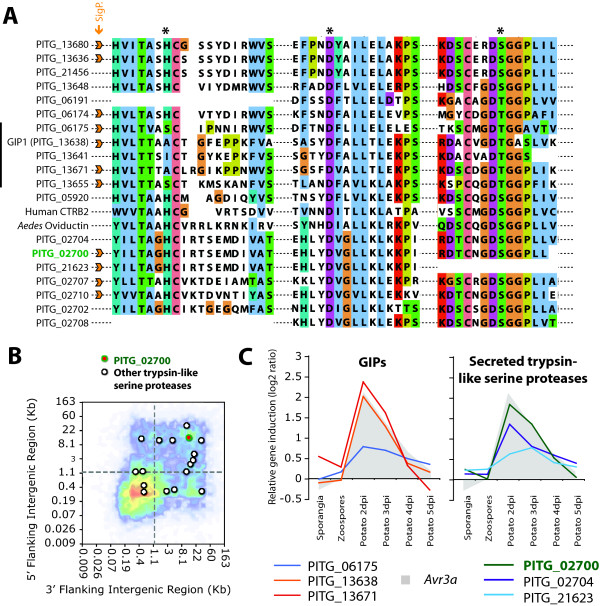
**PITG_02700: Trypsin-like serine protease**. **A**) Multiple sequence alignment showing the sequence similarity between PITG_02700 and its paralogs and well-characterized human and *Aedes *homologs. Regions spanning the catalytic triad (indicated by *) are shown. Proteins belonging to the *P. infestans *secretome are labeled with a signal peptide (SigP.) icon. GIP1, Glucanase Inhibitor Protein 1. **B**) Position of PITG_02700 and other *P. infestans *trypsin-like serine proteases on the FIR heat map (Figure 2B). **C**) *in planta *expression pattern of three *in planta*-induced GIP-like genes (left) and three other secreted serine protease genes (right), including PITG_02700. Expression of the effector gene *Avr3a *is given as a reference. Dpi, days post inoculation.

PITG_02700 encodes a predicted trypsin-like serine protease related to Glucanase Inhibitor Proteins (GIPs), which are catalytically inactive proteases that function as apoplastic effectors [9,13]. PITG_02700 belongs to a family of 19 paralogs in *P. infestans *among which 11 are predicted to be secreted (Figure 4A). Only two out of the 19 corresponding genes reside in GDRs (Figure 4B). Unlike the GIPs, the catalytic triad of PITG_02700 is intact suggesting a functional serine protease (Figure 4A). Similar to some GIP genes (Figure 4C), PITG_02700 and its closest paralogs PITG_02704 and PITG_21623 are induced *in planta *at 2 dpi (Figure 4D).

### Berberine bridge enzymes

**Figure 5:**
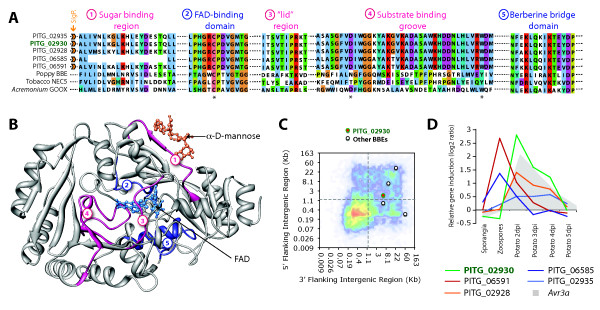
**PITG_02930: Berberine bridge enzyme**. **A) **Multiple sequence alignment showing the sequence similarity between PITG_02930 and its paralogs and well-characterized plant and fungal homologs. The FAD binding residues are indicated by *. Proteins belonging to the *P. infestans *secretome are labeled with a signal peptide (SigP.) icon. Aligned regions are numbered in the same way as in panel B to facilitate matching to the predicted protein structure. Regions indicated in blue show better conservation than regions in pink. **B) **Modeled protein structure of PITG_02930 with the regions shown in panel A highlighted. **C**) Position of PITG_02930 and other *P. infestans *BBEs on the FIR heat map of *P. infestans *(Figure 2B). **D**) *in planta *expression pattern of the five *P. infestans *BBEs. Expression of the effector gene *Avr3a *is given as a reference. Dpi, days post inoculation.

PITG_02930 has similarity to berberine bridge enzyme (BBE) genes. BBEs are flavoenzymes related to oligosaccharide oxidases found in archaea, bacteria, plants and fungi. They are involved in the generation of reactive oxygen species and in the synthesis of alkaloids in plants. Five BBE isoforms were predicted in the *P. infestans *genome, all of which harbor a predicted signal peptide. To gain insights into the impact of sequence polymorphisms on the activity of these enzymes, we aligned the BBE sequences to well characterized homologs from plants and fungi (Figure 5A, Additional file 7) and modeled the 3D structure of *P. infestans *BBEs (Figure 5B). All five *P. infestans *BBEs possess the three residues required for FAD cofactor binding in fungal glucooligosaccharide oxidases (GOOX, related to BBEs, Figure 5A) and show a good conservation of the FAD-binding and BBE domains compared to their plant and fungal counterparts (Figure 5A and 5B, '2' and '4'). Polymorphic residues within the *P. infestans *BBE clade are mostly found in the sugar-binding region (Figure 5A and 5B '1'). The substrate binding groove region of *Phytophthora *BBEs (Figure 5A and 5B '4') is divergent from BBEs in other species. The binding groove is widely open in fungal GOOX presumably to accommodate a range of substrates. In contrast, the binding groove in the *P. infestans *modeled BBE is largely obstructed by a coil of amino acids running from one side to the other of the binding pocket (Figure 5A and 5B, '3'). These observations suggest that *P. infestans *BBEs may have evolved to recognize a distinct set of substrates relative to their fungal and plant counterparts. *P. infestans *BBE genes are all excluded from GDRs (Figure 5C) and are either weakly (PITG_02935, PITG_06585) or strongly (PITG_02930, PITG_02928, PITG_06591) induced at 2 dpi *in planta *(Figure 5D).

### Alpha carbonic anhydrases

**Figure 6:**
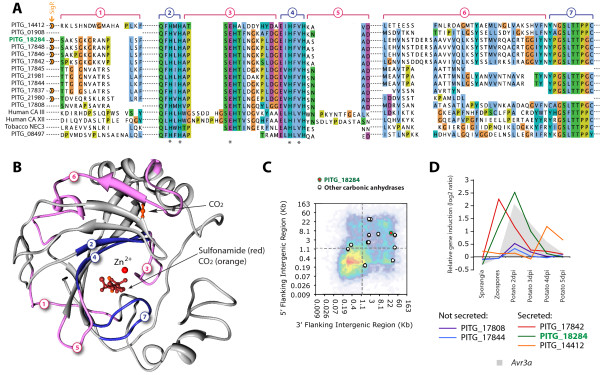
**PITG_18284: Alpha-carbonic anhydrase**. **A) **Multiple sequence alignment showing the sequence similarity between PITG_18284 protein from the plastic secretome and its paralogs and well-characterized plant and human homologs. The CO_2 _binding residues are indicated by *. Proteins belonging to the *P. infestans *secretome are labeled with a signal peptide (SigP.) icon. Aligned regions are numbered in the same way in panel B to facilitate matching the sequence to the predicted protein structure. Regions indicated in blue show better conservation than regions in pink. **B) **Modeled protein structure of PITG_18284 with the regions shown in panel A highlighted. **C**) Position of PITG_18284 and other *P. infestans *α-CA on the FIR heat map of *P. infestans *(Figure 2B). **D**) *in planta *expression pattern of five *P. infestans *α-CAs. Non-secreted α-CAs are not induced *in planta *(PITG_17808 and PITG_17844), whereas secreted α-CAs show early (PITG_17842 and PITG_18284) or late induction (PITG_14412). Expression of the effector gene *Avr3a *is given as a reference. Dpi, days post inoculation.

PITG_18284 was annotated as an alpha-carbonic anhydrase (α-CA). The *P. infestans *genome encodes 13 predicted α-CAs among which seven belong to the secretome. To explore the structural properties of the *P. infestans *α-CAs, we aligned their sequences to the closest human homologs and to tobacco NEC3 α-CA (Figure 6A, Additional file 7), and modeled the 3D structure of PITG_18284 and PITG_17842 (Figure 6B). When compared to human and tobacco homologs, *P. infestans *α-CAs show a conserved core surrounding the active site (Figure 6A and 6B, '2' SEHT motif of '3', '4' and '7') with conserved catalytic residues (with the exception of PITG_08497). On the contrary, regions at the surface of the enzyme are variable between *P. infestans *α-CAs and differ from human and tobacco enzymes ('1', '5' and '6', residues surrounding the SEHT motif of '3'). This notably results in the absence in the *P. infestans *enzymes of an alpha helix gating the entry of the zinc-binding pocket in human enzymes. Residues in this alpha helix are in close proximity with sulfonamide inhibitor in human models suggesting that *P. infestans *α-CAs may have evolved alternative docking properties at the entrance of the zinc-binding groove. All *P. infestans *α-CA genes, except PITG_08497, are excluded from GDRs (Figure 6C). Whereas the *P. infestans *α-CA genes that encode non-secreted enzymes are not induced *in planta *(PITG_17808 and PITG_17844 in Figure 6D), most of the genes encoding secreted α-CAs are strongly induced either early (PITG_17842 and PITG_18284) or late (PITG_14412) during plant infection (Figure 6D).

### Novel small cysteine-rich (SCR) proteins

**Figure 7:**
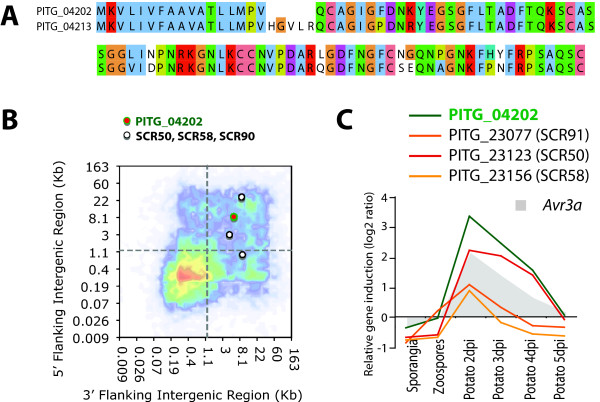
**PITG_04202: Small cysteine rich proteins (SCR)**. **A**) Pairwise sequence alignment of SCR PITG_04202 and its closest paralog. **B**) Position of PITG_04202 and known SCRs genes on the FIR heat map of *P. infestans*. **C**) *in planta *expression pattern of known SCR genes (SCR58, SCR91 and SCR50) and PITG_04202. Expression of the effector gene *Avr3a *is given as a reference. Dpi, days post inoculation.

Many filamentous pathogen effectors encode small (<150 amino acids) secreted proteins with an even number of cysteine residues that form disulfide bridges [5]. We found 265 small (50 to 150 amino-acids) cysteine-rich (>5% of sequence) *in P. infestans *(Additional file 8). Among them, 59 are predicted to be secreted, 17% of which are induced *in planta *(Additional file 8). In particular, PITG_04202 is a gene from the plastic secretome that encodes a 94 amino acid SCR with six cysteines (Figure 7). It has one close paralog (PITG_04213) that encodes a 99 amino acid protein with the six cysteine residues conserved. PITG_04202 is induced *in planta *during the biotrophic phase similar to previously studied SCR effectors such as SCR91, SCR50, and SCR58.

### Repeat containing proteins (RCPs)

**Figure 8:**
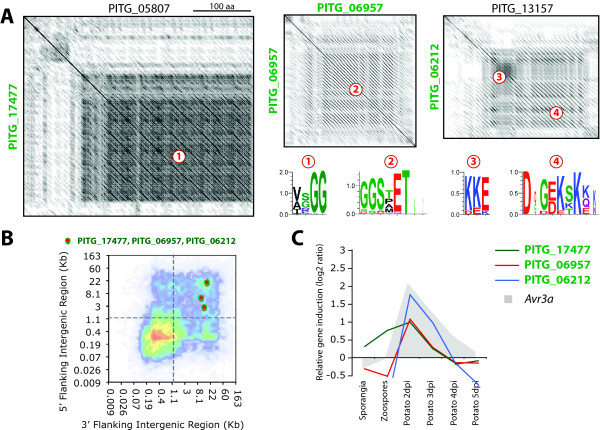
**PITG_17477, PITG_06957, and PIG_06212: Repeat containing proteins (RCPs)**. **A**) Sequence identity dot plots showing internal amino-acid sequence repeats found in PITG_17477, PITG_06957, PIG_06212 (in green) and their closest paralogs (except for PITG_06957, which lack paralogs). Numbers refer to MEME amino-acid motifs found within the repeats as indicated. **B**) Position of RCP genes on the FIR heat map of *P. infestans*. **C**) *in planta *expression pattern of the RCP genes. Expression of the effector gene *Avr3a *is given as a reference. Dpi, days post inoculation.

Many microbial adhesins are repetitive proteins with different types of repeats, such as glycine-rich repeats. Some oomycete repeat containing proteins are secreted proteins that are thought to function in adhesion, and include *P. infestans *mucin-like protein CAR90 [56], IPIB [44], and M96 mating-specific proteins [57]. Several of the *P. infestans *genes from the plastic secretome that are induced *in planta *encode repeat-containing proteins not described to date. PITG_17477 encodes a 374 amino acid protein with more than 30% glycine residues due to 48 [VA][GS]GG repeats. It has one close paralog in *P. infestans*, PITG_05807 (Figure 8A). The PITG_17477 gene is induced during the biotrophic phase of potato infection (Figure 8B).

PITG_06957 encodes a 247 amino acid protein with 53 glycine residues organized in 22 imperfect GGSxET repeats (Figure 8A). This gene lacks paralogs in *P. infestans*, and this class of repeats is absent from other *P. infestans *proteins. PITG_06957 is induced two-fold during the biotrophic phase of potato infection (Figure 8B).

Besides Glycine-rich repeat containing proteins, PITG_06212 is a 232 amino acid protein that contains 64 lysine residues organized in 11 KKE repeats followed by 10 DxGEKSKKx repeats (Figure 8A). The same repeat pattern was observed in the sequence of the protein encoded by the paralogous gene PITG_13157. PITG_0621 is induced during the biotrophic phase of potato infection (Figure 8B).

## Discussion

We exploited genome organization to augment other criteria for selection of candidate virulence genes in the oomycete plant pathogen *P. infestans*. Based on the work of Haas *et al. *(2009), genome organization appears to be a good indicator of virulence genes in *P. infestans*. Can this strategy be extended to explore and identify novel effectors from other pathogens? Effector genes often occur in plastic genomic regions. A remarkable example is the plant pathogenic fungus *Leptosphaeria maculans *in which the *AvrLm1, AvrLm6 and AvrLm4-7 *effector genes reside in 100 kb or larger AT-rich gene-poor isochores [58-60]. In other plant pathogenic fungi, such as *Alternaria alternata *[61], *Mycosphaerella graminicola *[62], and *Fusarium graminearum *[63], some effector genes are carried in conditionally dispensable chromosomes. Localization of effectors in plastic genome regions also extends to animal pathogens. Host-translocated effectors from *Plasmodium *are often found near telomeric regions of chromosomes [25]. These specific effector genome niches in eukaryotic pathogens are reminiscent of the highly variable bacterial pathogenicity islands that carry clustered translocation machinery and effector genes [64]. In summary, localization of effector genes to dedicated plastic regions of pathogen genomes is a frequent occurrence. The strategy we applied in this work enabled the identification of previously overlooked candidate virulence genes and is in principle applicable to a wide range of eukaryotic pathogenic microorganisms.

Plastic genome regions can take several forms such as dispensable chromosomes or telomeric regions. Are there conserved features that characterize plastic genome regions? How can we recognize them? High density of active mobile DNA transposable elements (TEs) can be considered a signature of variable genome regions. TEs have long been considered "selfish genes" for causing chromosomal breaks, deletions, or translocations [65]. But several studies now show that TEs are major drivers of rapid evolution and functional diversification of gene families [66] as well as evolution of gene regulation [67,68]. TEs tend to accumulate around genes involved in stress response, defense and response to external cues [66]. The length of the intergenic regions flanking each gene reflects the impact of TEs on local gene density. Analysis of the distribution of FIRs helps to visualize localized and differential TE activity and to identify plastic genome regions [15]. In this regard, *P. infestans *stands out by its dramatic uneven distribution in FIR lengths that results in a clear demarcation of GDRs vs GSRs (Figure 2B). This extreme property of the *P. infestans *genome allowed us to quantify the degree of association between effector genes and plastic genome regions. Clearly, effector genes almost exclusively reside in GSRs, supporting a contribution of TE activity to effector evolution (Figure 2D).

Among the novel candidate virulence genes we identified, there were two types of oxidoreductases (berberine-bridge enzyme and alpha-carbonic anhydrase). The presence of enzymes catalyzing conversion of rather simple molecules within the plastic secretome of *P. infestans *is perhaps surprising. What role may such catalytic enzymes play in the interaction between *P. infestans *and host plants? How do polymorphisms in these enzymes affect host interactions? BBEs are flavoenzymes that catalyze carbohydrate oxidation in plants, either for the biosynthesis of berberine type alkaloids, or for the generation of hydrogen peroxide (H_2_O_2_). Plant BBEs are highly induced during various defense responses, when they may contribute to the oxidative burst leading to cell death, through H_2_O_2 _synthesis. CAs typically function in acid-base balance control by rapidly converting carbon dioxide to bicarbonate. CA activity is also required for the onset of disease resistance in tobacco. Silencing of a CA gene in the plant *Nicotiana benthamiana *results in enhanced susceptibility to *P. infestans *[69] and a salicylic acid binding protein SABP3 exhibiting CA activity is required for the onset of the hypersensitive response toward the bacterial plant pathogen *Agrobacterium tumefaciens *[70]. Therefore oxidoreductases might be involved in triggering or enhancing host cell death responses during the necrotrophic phase of *P. infestans *growth. Alternatively, H_2_O_2 _production may contribute to plant cell wall degradation by *P. infestans*. The ability to degrade alkaloids may also contribute to virulence of various plant pathogens [71], for instance by counteracting antimicrobial properties of plant-synthesized alkaloids (such as berberine) and sulfonamides (such as quinine, potent inhibitors of α-CAs) [72,73]. In any case, it is possible that evasion of plant inhibitors (e.g. plant-specific sulfonamides) contributes to rapid evolution in *P. infestans *secreted BBE and α-CA enzymes. Plant secondary metabolites are structurally highly diverse, and their corresponding biosynthetic genes are frequently associated with divergent genome regions [74,75]. Plant-pathogen arms race coevolution might result in a parallel highly divergent detoxification arsenal in pathogen genome. The examples of BBE and α-CA described here emphasize the need for integrated metabolomic surveys of plant-pathogen interactions.

Cell wall degrading enzymes (CWDEs) are a hallmark of filamentous pathogen secretomes [26,76,77]. A diverse repertoire of secreted CWDEs matches the variety of sugar polymers that make up plant cell walls. Two *P. infestans *genes from the plastic secretome, PITG_02524 and PITG_08563, are predicted pectin lyases, which are known in other pathogens as virulence factors that degrade the pectic components of plant cell walls [78]. Another gene from the plastic secretome, PITG_22758, is related to concanavalin A lectins/glucanases, which carry out the acid catalysis of beta-glucans [79] or function in cell recognition in eukaryotes [80]. In plants, lectins show a wide variety of protein structures and sugar binding properties that matches the diversity of sugar molecules [81]. It is therefore reasonable to correlate the diversity of *P. infestans *secreted CWDEs to the complexity of the plant cell wall. But how to explain the high divergence observed in the CWDEs in plastic regions? First, plant cell walls are highly variable from one plant species to another and between different stages of plant development [82]. Therefore secreted CWDEs genes residing in plastic genome regions may have enabled faster adaptation to a new host or tissue (for instance, leaf vs root). Second, plants have evolved a number of CWDE inhibitors as a pathogen defense mechanism [83]. Rapid evolution in *P. infestans *secreted CWDEs may have been driven by arms race coevolution with host inhibitors. Third, cell wall degradation products can act as damage-induced molecular patterns (DAMPs) and trigger plant immune responses [84]. *P. infestans *CWDEs may therefore evolve to minimize DAMP induction. In summary, localization of particular carbohydrate binding protein genes in plastic genomic regions may have contributed to the pathogenic success of *P. infestans*.

It is well accepted that due to metabolic costs and spatial constraints, genome expansion is globally selected against unless it provides an important functional advantage [85]. Although evidence for the contribution of non-coding DNA expansion to gene evolution continues to accumulate, the mechanisms that enable faster gene evolution remain poorly understood. Unlike housekeeping genes, most effector genes show a "patchy" phylogenetic distribution, being present in *P. infestans *but lacking in *P. sojae *and *P. ramorum*. Similar properties are typical of the virulence genes of a variety of fungal and oomycete pathogens [6,86]. This can be due to high rates of mutations, gene loss, copy number variation (CNV), or horizontal gene transfer that are thought to occur more frequently in plastic regions of the genome. One example is the large specific deletion spanning *AvrLm1 *that is responsible for gain of virulence on *Rlm1 *plants in *L. maculans *[23]. Similar gene deletions were reported for several fungal plant pathogen avirulence loci, such as *Avr9 *and *avr4E *of *Clasdosporium fulvum *[87], *SIX1 *of *Fusarium oxysporum *[88] and *Avr1-CO39 *and *Avr-Pita *of *Magnaporthe grisea *[89,90]. Additionally, an excess of CNV and increased sequence polymorphisms were noted toward chromosomal ends in *Plasmodium *spp. [91]. Such genome remodeling might preferentially occur in regions with extensive non-coding DNA because of reduced deleterious consequences to cis-linked genes [92]. Another hypothesis is that longer flanking regions enable the development of more tightly and accurately regulated expression patterns [65,92], possibly through epigenetic variation [90,93]. Future comparative genomics of clusters of closely related pathogen species will help to further clarify the mechanisms underlying rapid evolution of plastic genome regions and to test these various hypotheses.

## Conclusions

In this study, we predicted and annotated the secretome of the Irish potato famine pathogen *P. infestans *using *in silico *approaches. We quantitatively described *P. infestans *genome organization by delimiting gene dense and gene sparse regions. We used genome organization as a novel approach that augments previously established criteria to mine for candidate virulence factors. Occurrence of secreted protein genes in GSRs, in combination with comparative genomics and transcriptomics, implicated 19 previously overlooked genes in virulence. These include cell wall degrading enzymes, trypsin-like serine protease, carbonic anhydrase, berberine bridge enzyme, several repeat containing proteins, and small cysteine-rich proteins.

## Methods

### Identification of putative secreted proteins

Signal peptide predictions were performed following the methods of Torto *et al. *(2003) [30] and Win *et al. *[19]. The 18,155 proteins predicted by Haas *et al. *(2009) [15] from the *P. infestans *T30-4 genome assembly were submitted to SignalP v2.0 [94]. A SignalP HMM score cutoff of ≥ 0.9 was used (2,228 proteins recovered). This set of 2228 proteins was submitted to SignalP3.0 [95], RPSP [96], TargetP [97], WolfPSort [98] and TMHMM [99] (Additional file 1). Proteins showing (i) SignalP2.0 HMM score ≥ 0.9 and (ii) SignalP3.0 NN Ymax Score ≥ 0.5 and (iii) SignalP3.0 NN D-score ≥ 0.5 and (iv) SignalP3.0 HMM S probability ≥ 0.9 and (v) TargetP predicted localization "Secreted" (S) and (vi) most probable PSort location "extracellular" (extr.) and no TMHMM predicted transmembrane domain after signal peptide cleavage site were considered as *P. infestans *secretome.

### Enrichment analyses

Pfam [100] and Superfamily 1.73 [101] with default parameters were used to complement the annotation of the secreted proteins. Gene Ontology (GO) terms mapping was performed on *P. infestans *proteome using Blast2GO [102] with default parameters and GO sorted by domain (Additional file 1). The number of occurrences of each Pfam domain, Molecular function GO and Biological process GO found in secretome was calculated among secretome proteins and the rest of the proteome. Frequencies are given as the number of occurrences over the total number of Pfam domain or GO hits among secreted or non-secreted proteins. Enrichment fold correspond to frequency in secretome over frequency in the rest of the proteome. Depletion fold (1 over enrichment fold) is given for domains/ontologies depleted from secretome. Significance of enrichment/depletion is assessed by a chi-square test with Bonferroni correction for multiple testing. Only Pfam domains with enrichment p-value ≤ 0.1 and at least one hit with e-value ≤ 10e^-05 ^and GO with enrichment p-value ≤ 0.1 are reported in figure 1.

### Identification of genes belonging to orthologous segments

Genes belonging to orthologous segments were identified in Haas et al. [15]. Briefly, regions of conserved collinear gene order between *P. infestans*, *P. sojae *and *P. ramorum *genomes were computed using DAGchainer 30 considering only the relative order of the genes along each scaffold [103]. Only orthologs defined by OrthoMCL 24 [104] were used as anchors for collinear blocks. Collinear blocks were defined between each pair of the three *Phytophthora *genomes. The orthologous segments reported corresponds to the union of blocks obtained from the pairwise comparisons to the other genomes.

### Sequences alignments

Similarity searches were performed using Blastall from NCBI Blast package [105]. Sequences were aligned using Clustal W2 program [106], rendered with Jalview [107] and manually annotated. Protein domains in candidate virulence genes were identified using Pfam [100]. Identity dotplots for Repeat containing proteins were drawn using Dotlet with word size of 7 [108], motifs were found using MEME [109].

### Gene expression analysis

Whole genome expression data used in this work were previously described by Haas *et al. *[15] and are based on a custom NimbleGen oligonucleotide microarray. *P. infestans *genes were classified as induced when they showed at least a 2-fold induction during colonization of potato at 2, 3, 4 or 5 days post inoculation (dpi), or tomato at 2 or 5 dpi, compared to *in vitro *grown mycelia. In Figures 4, 5, 6, 7 and 8, gene expression is given as log_2 _(linear expression in sample/average linear expression in control mycelia).

### Protein 3D modeling and structure analysis

3D structure of PITG_02935, PITG_02930 and PITG_06585 *P. infestans *BBEs were modeled based on homology with the template protein structures of *Acremonium strictum *1ZR6A [110] and *Eschscholzia californica *3D2H [111]. The align2d function and 3D modeling in modeler9v7 [112] were used for that purpose. 3D structure of PITG_17842 and PITG_18284 α-CA were predicted using similar methods by homology with human 1FLJA [113] and 1JD0A [114]. Rendering of the models was performed with Chimera [115]. To compare protein structures, the models were superimposed by matching C, N and O atoms from residues H94, H96, H119 of 1JD0.A to H92, H94, H111 of PITG_18284 model; C130, D355, W383 of 1ZR6 to C146, D373, W401 of PITG_02930 model; C166, W328, I516 of 3D2D to C146, W311, I487 of PITG_02930 model.

## Abbreviations

BBE: Berberine Bridge Enzyme; CA: Carbonic Anhydrase; CNV: Copy Number Variation; CRN: Crinkler effector; CWDE: Cell Wall Degrading Enzyme; DAMP: Damage induced Molecular Pattern; dpi: days post inoculation; FIR: Flanking Intergenic Region; GDR: Gene Dense Region; GIP: Glucanase Inhibitor Protein; GOOX: Glucooligosaccharide oxidase; GSR: Gene Sparse Region; OS: Orthologous Segments; RCP: Repeat-Containing Protein; SCR: Small Cysteine-Rich protein; TE: Transposable Element.

## Authors' contributions

SR carried out most analyses, contributed to the design of the study and drafted the manuscript. JW performed the secretome predictions, provided guidance with some of the analyses and contributed to the writing of the manuscript. LC helped with the gene expression analyses and contributed to the writing of the manuscript. SK conceived and designed the study, edited the manuscript and supervised the work. All authors read and approved the final manuscript.

## Supplementary Material

Additional file 1 **Complete list of *P. infestans *predicted secretome**. Microsoft Excel spreadsheet containing complete list of *P. infestans *secretome genes, including annotation steps, biological processes and biological function categories, genomic and expression data. Click here for file

Additional file 2 **The distribution of *P. infestans *complete proteome and secretome according to protein length, cysteine and glycine content**. Graphs showing the distribution of protein length, cysteine and glycine content among *P. infestans *genes. Click here for file

Additional file 3 **Gene conservation in *P. infestans *plastic secretome**. A) The different levels of orthology and conservation for *P. infestans *genes. 5994 *P. infestans *genes are absent from genome segments orthologous with *P. sojae *and *P. ramorum *genomes, 2396 of them do not have orthologs (1.e-^10 ^blastP e-value cutoff) in *P. sojae *or *P. ramorum *genomes. B) Frequency of *P. infestans *genes with orthologs in *P. sojae *and *P. ramorum*. The level of orthology is given by % of sequence identity between *P. infestans *protein and its best BlastP hit in *P. sojae *or *P. ramorum *proteome. Frequencies among non-secreted proteins (grey), plastic secretome (green) and other secretome proteins (yellow) are shown. Click here for file

Additional file 4 **Frequency of secretome genes, genes from non-OS, GDR and GSRs**. table showing the number and % of genes from various groups matching specific conditions related to genome environment and secretome features Click here for file

Additional file 5 **Number and frequency of genes induced *in planta***. table comparing the distribution of genes induced *in planta *at different time point from the whole genome and the plastic secretome Click here for file

Additional file 6 **Genomic context of the 19 genes from the plastic secretome induced *in planta *described in details in this manuscript**. Figure showing genes, orthologous gene pairs, and repeated sequences in the genomic environment of 19 genes from the plastic secretome. Click here for file

Additional file 7 **Alignments used for 3D modeling of *P. infestans *berberine bridge enzymes and alpha carbonic anhydrase**. Click here for file

Additional file 8 **Global analysis of *P. infestans *Small Cysteine-rich proteins**. A) Distribution of *P. infestans *proteins according to their length and cysteine content. B) Position of PITG_04202 and other *P. infestans *secreted SCRs on the FIR heat map. C) Frequency of SCR sequences as a % of *P. infestans *whole proteome, secretome and non-secreted proteins; frequency of SCR genes induced as a % of SCR genes in *P. infestans *whole genome, secretome and non-secretome genes. Click here for file
