# Supplementary material for: Analyses of genome architecture and gene expression reveal novel candidate virulence factors in the secretome of Phytophthora infestans
Source: BMC Genomics. 2010 Nov 16;11:637. doi: 10.1186/1471-2164-11-637 (PMC3091767; doi:10.1186/1471-2164-11-637)
Supplement: Additional file 2 — The distribution of P. infestans complete proteome and secretome according to protein length, cysteine and glycine content. Graphs showing the distribution of protein length, cysteine and glycine content among P. infestans genes. [file 1471-2164-11-637-S2.PDF]

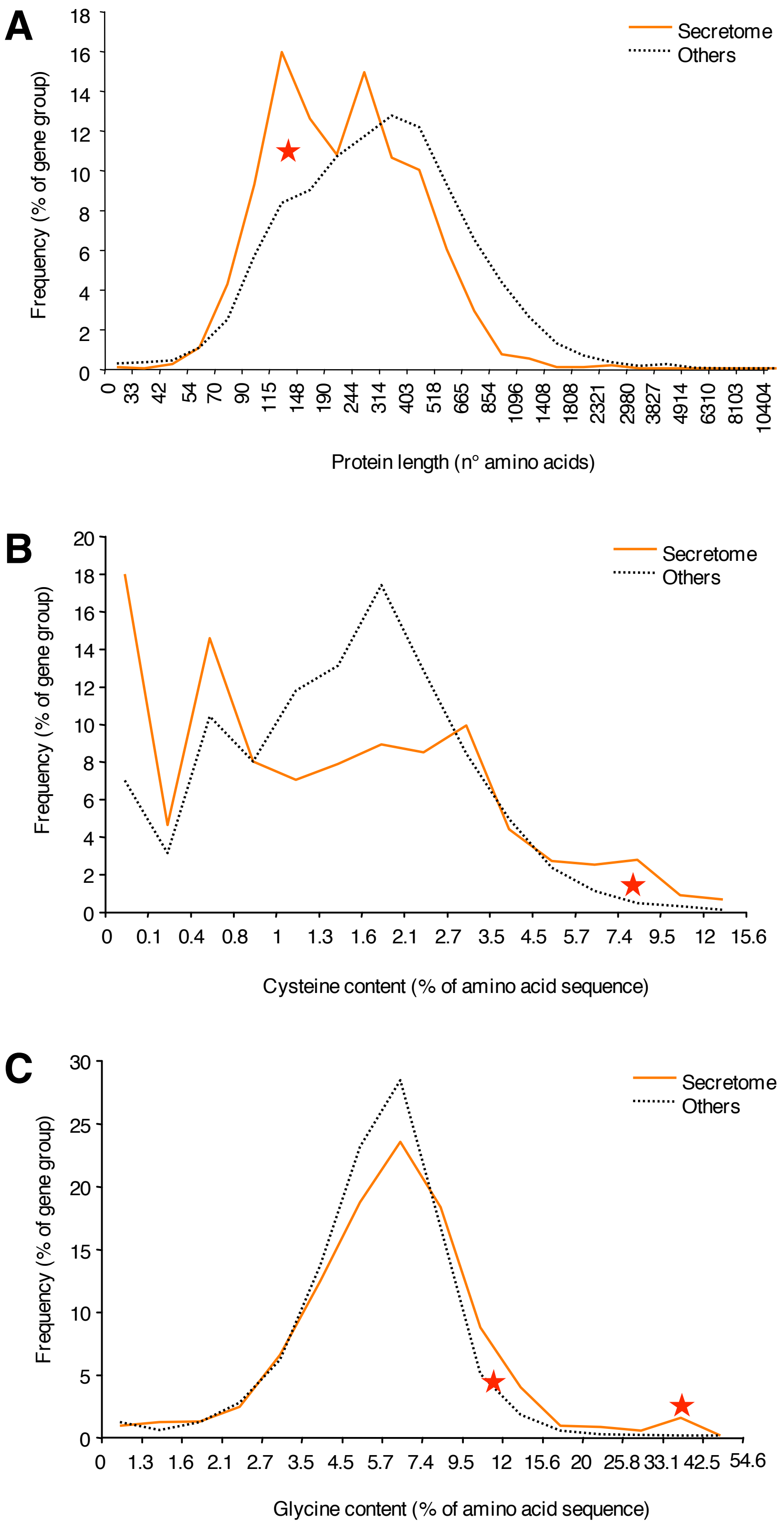

Additional file 2. The distribution of *P. infestans* secretome (plain, orange) and non secreted proteins (dotted, black) according to protein length (A), cysteine (B) and glycine content (C).
