# Supplementary material for: Analyses of genome architecture and gene expression reveal novel candidate virulence factors in the secretome of Phytophthora infestans
Source: BMC Genomics. 2010 Nov 16;11:637. doi: 10.1186/1471-2164-11-637 (PMC3091767; doi:10.1186/1471-2164-11-637)
Supplement: Additional file 3 — Gene conservation in P. infestans plastic secretome. A) The different levels of orthology and conservation for P. infestans genes. 5994 P. infestans genes are absent from genome segments orthologous with P. sojae and P. ramorum genomes, 2396 of them do not have orthologs (1.e-10 blastP e-value cutoff) in P. sojae or P. ramorum genomes. B) Frequency of P. infestans genes with orthologs in P. sojae and P. ramorum. The level of orthology is given by % of sequence identity between P. infestans protein and its best BlastP hit in P. sojae or P. ramorum proteome. Frequencies among non-secreted proteins (grey), plastic secretome (green) and other secretome proteins (yellow) are shown. [file 1471-2164-11-637-S3.PDF]

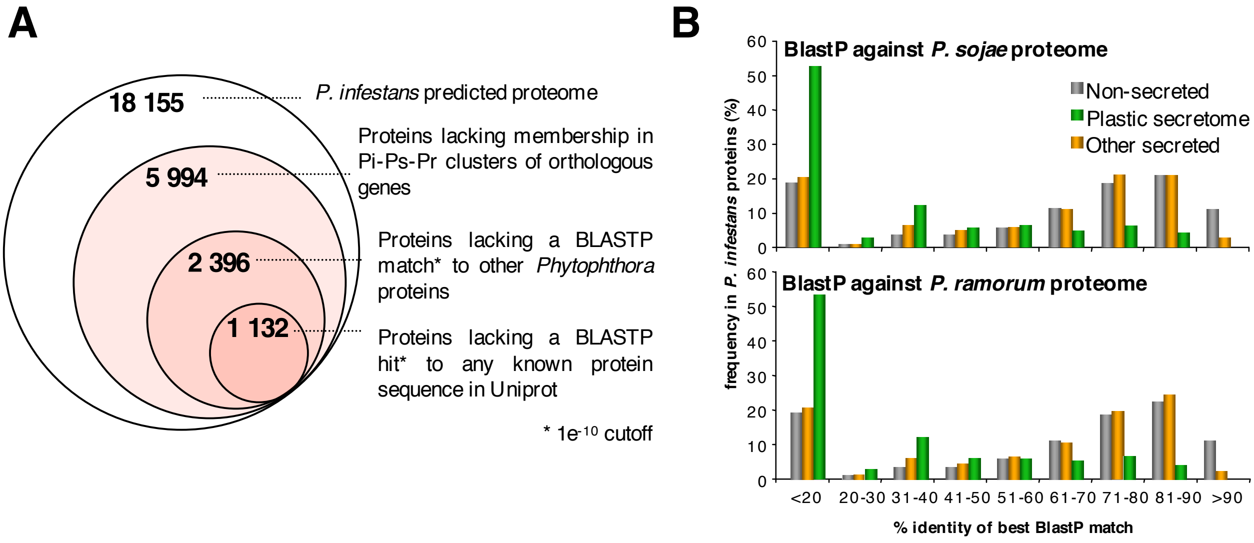

Additional file 3. Orthology relationship between *P. infestans, P. sojae* and *P. ramorum* genes

A) The different levels of orthology and conservation for *P. infestans* genes. 5994 *P. infestans* genes are absent from genome segments orthologous with *P. sojae* and *P. ramorum* genomes, 2396 of them do not have orthologs (1.e-^{10} blastP e-value ciutoff) in *P.sojae* or *P. ramorum* genomes.

B) Frequency of *P. infestans* genes with orthologs in *P.sojae* and *P. ramorum*. The level of orthology is given by % of sequence identity between *P. infestans* protein and its best BlastP hit in *P. sojae* or *P. ramorum* proteome. Frequencies among non-secreted proteins (grey), plastic secretome (green) and other secretome proteins (yellow) are shown.
