# Supplementary material for: Analyses of genome architecture and gene expression reveal novel candidate virulence factors in the secretome of Phytophthora infestans
Source: BMC Genomics. 2010 Nov 16;11:637. doi: 10.1186/1471-2164-11-637 (PMC3091767; doi:10.1186/1471-2164-11-637)
Supplement: Additional file 4 — Frequency of secretome genes, genes from non-OS, GDR and GSRs. table showing the number and % of genes from various groups matching specific conditions related to genome environment and secretome features [file 1471-2164-11-637-S4.DOC]

**Additional file 4. Frequency of various gene groups in secretome, in genome regions, in orthologous segments (OS) and in plastic secretome.** Shown are % of the gene group indicated in first column matching condition indicated in the first line. In the case of effector genes, the % of effector genes in validated secretome and matching a specific condition is indicated.

|  | Total | In validated Secretome | In GSRs  (2 FIRs≥1.5Kb) | In “Between” (1 FIR≥1.5Kb) | In GDRs  (2 FIRs<1.5Kb) | Excluded from OS^a^ | In plastic secretome |
| --- | --- | --- | --- | --- | --- | --- | --- |
| Core ortholog set^a^ | 7986 | 345  (4.3%) | 705  (8.8%) | 2939 (36.8%) | 4181 (52.4%) | 0  (0.0%) | 0  (0.0%) |
| Not secreted | 16741 | 0  (0.0%) | 3332 (19.9%) | 5743 (34.3%) | 6445 (38.5%) | 7018 (41.9%) | 0  (0.0%) |
| Secretome | 1415 | 1415 (100.0%) | 698 (49.3%) | 473 (33.4%) | 244 (17.2%) | 930 (65.7%) | 561 (39.6%) |
| Known effectors | 796 | 643 (80.8%) | 524 (65.8%) | 233 (29.3%) | 39  (4.9%) | 573 (89.1%) | 398 (61.9%) |
| Secretome non RXLR, non CRN | 904 | 904 (100.0%) | 337 (37.3%) | 336 (37.2%) | 231 (25.6%) | 450 (49.8%) | 220 (24.3%) |
| RxLR | 540 | 482 (89.2%) | 379 (70.2%) | 147 (27.2%) | 14  (2.6%) | 452  (93.8%) | 325 (67.4%) |
| CRNs | 132 | 29  (21.9%) | 77  (58.3%) | 49  (37.1%) | 6  (4.5%) | 28 (96.6%) | 16  (55.2%) |
| Genome | 18155 | 1415 (7.8%) | 4030 (22.2%) | 6216 (34.2%) | 6689 (36.8%) | 7948 (43.8%) | 561  (3.1%) |

FIR, Flanking Intergenic Region; GDR, Gene Dense Region; GSR, Gene Sparse Region; OS, Orthologous Segments. ^a^ Core ortholog sets and orthologous segments defined as in Haas *et al*. 2009.
