# Supplementary material for: Analyses of genome architecture and gene expression reveal novel candidate virulence factors in the secretome of Phytophthora infestans
Source: BMC Genomics. 2010 Nov 16;11:637. doi: 10.1186/1471-2164-11-637 (PMC3091767; doi:10.1186/1471-2164-11-637)
Supplement: Additional file 5 — Number and frequency of genes induced in planta. table comparing the distribution of genes induced in planta at different time point from the whole genome and the plastic secretome [file 1471-2164-11-637-S5.DOC]

**Additional file 5. Number and frequency of *in planta* induced genes**

| Genes induced ≥2-fold | Total  (% of whole genome) | In plastic secretome  (% of plastic secretome) | not RXLR not CRN In plastic secretome  (% of not RXLR not CRN plastic secretome) |
| --- | --- | --- | --- |
| Potato  2dpi | 827  (4.4%) | 88  (15.7%) |  |
| Tomato  2dpi | 369  (1.9%) | 49  (8.8%) |  |
| Potato  3dpi | 331  (1.7%) | 40  (7.1%) |  |
| Potato  4dpi | 177  (0.9%) | 14  (2.5%) |  |
| Tomato  5dpi | 94  (0.5%) | 16  (2.9%) |  |
| Potato  5dpi | 35  (0.2%) | 0  (0.0%) |  |
|  |  |  |  |
| Any of these time points | 1073  (5.8%) | 95  (17.0%) | 31  (19.0%) |

Dpi, days post inoculation.
