# Supplementary material for: Analyses of genome architecture and gene expression reveal novel candidate virulence factors in the secretome of Phytophthora infestans
Source: BMC Genomics. 2010 Nov 16;11:637. doi: 10.1186/1471-2164-11-637 (PMC3091767; doi:10.1186/1471-2164-11-637)
Supplement: Additional file 6 — Genomic context of the 19 genes from the plastic secretome induced in planta described in details in this manuscript. Figure showing genes, orthologous gene pairs, and repeated sequences in the genomic environment of 19 genes from the plastic secretome. [file 1471-2164-11-637-S6.PDF]

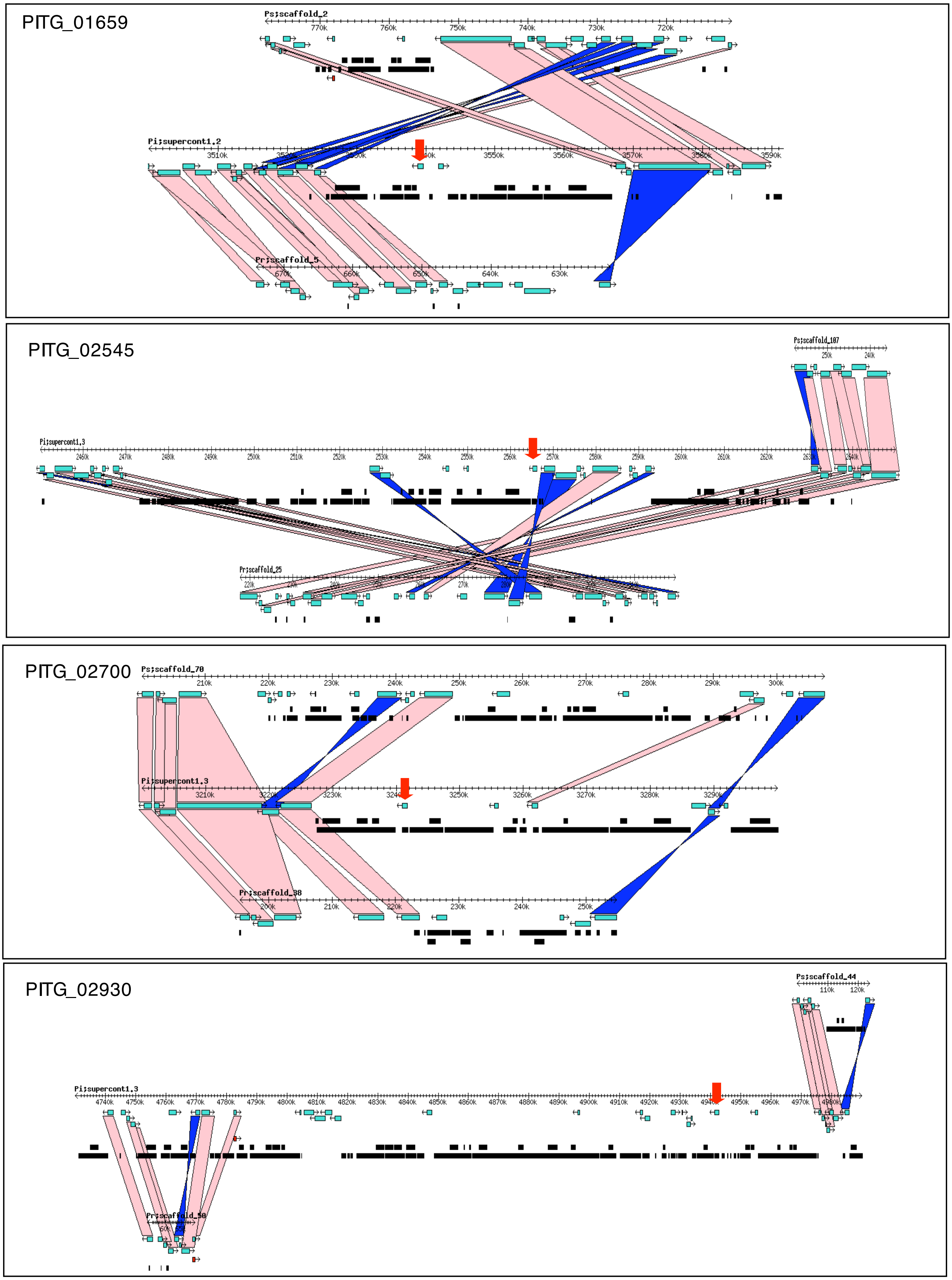

Additional file 6. Genomic context of the 19 plastic secretome genes induced in planta described in details in this manuscript. Show are genes (green arrows), orthologous gene pairs of same orientation (pink) or opposite orientation (blue) , repeatedd sequences (black). Plastic secretome genes are highlighted by a red vertical arrow.

Ps, *Phytophthora sojae*; Pi, *Phytophthora infestans*; Pr, *Phytophthora ramorum*.

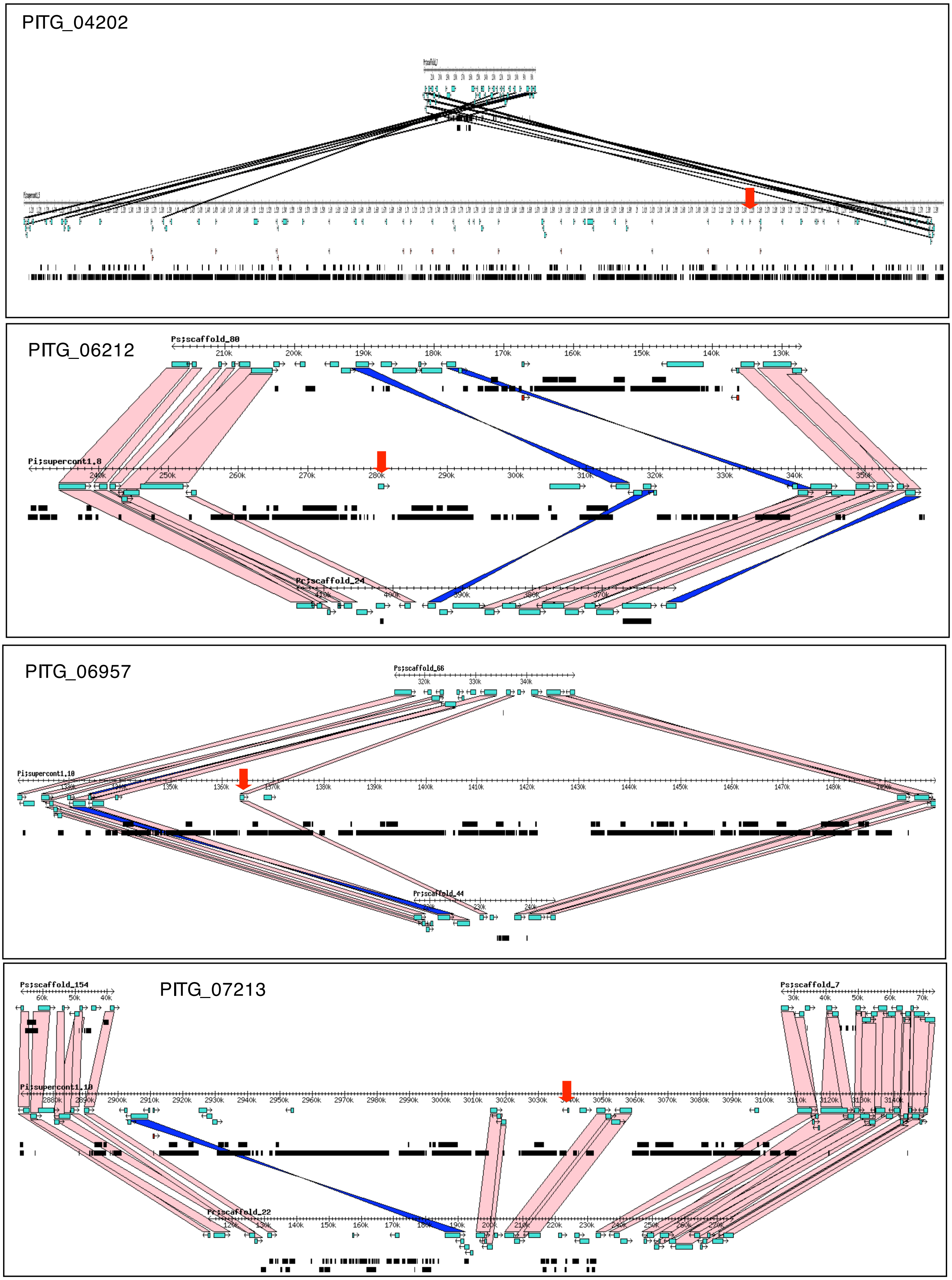

Figure S3 continued

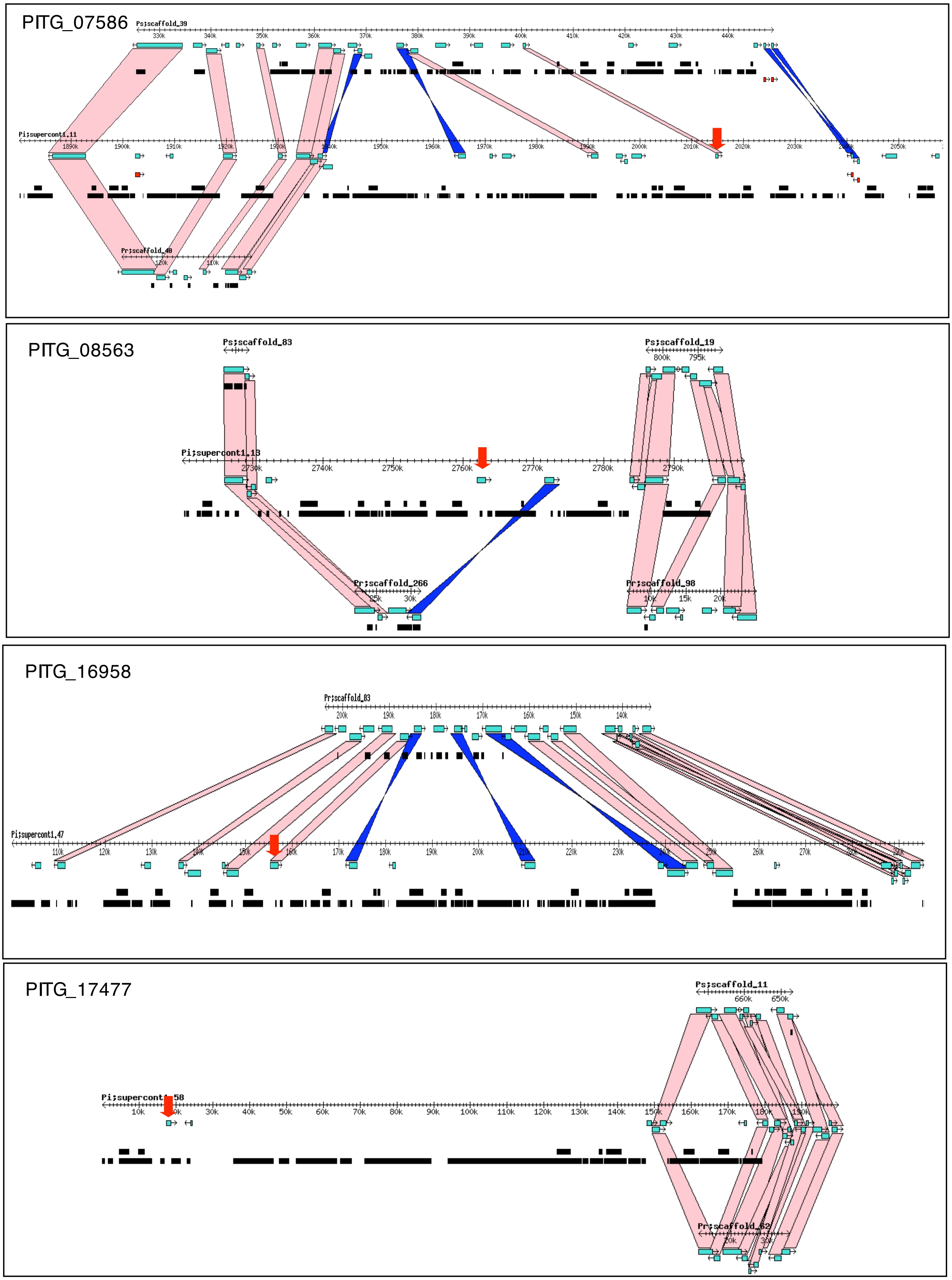

Figure S3 continued

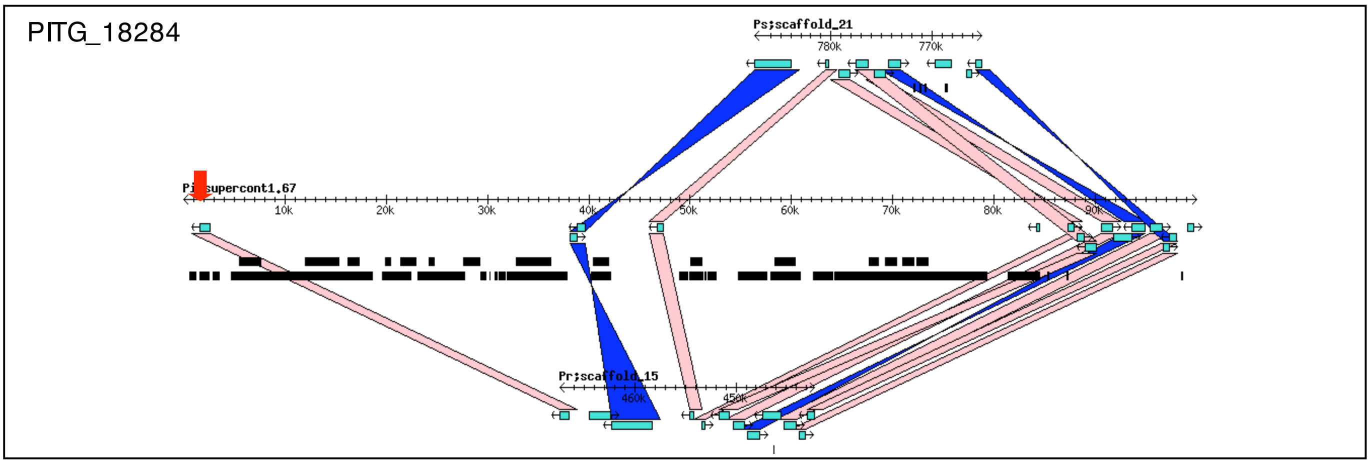

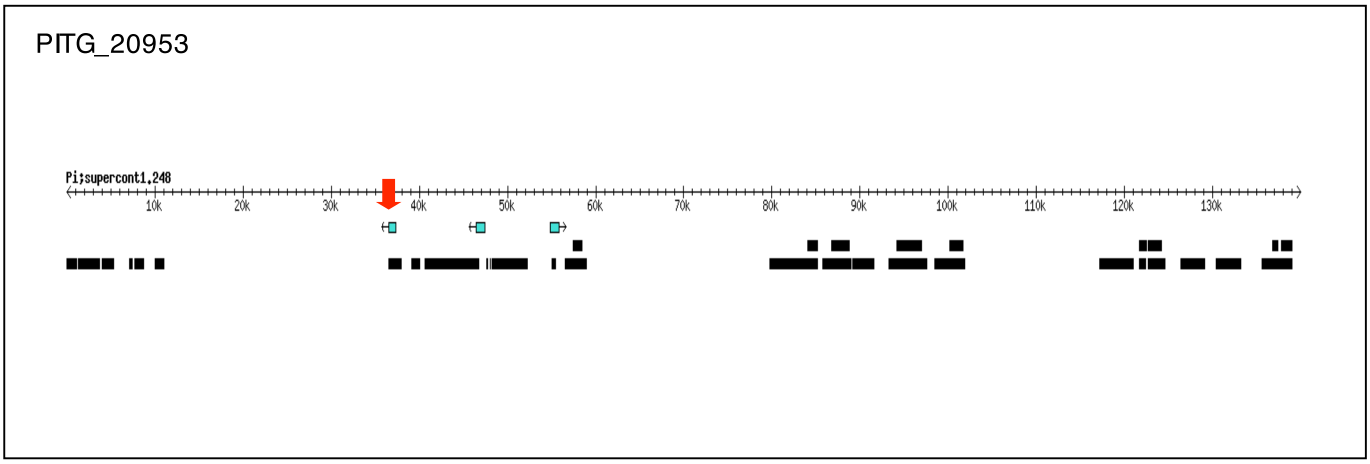

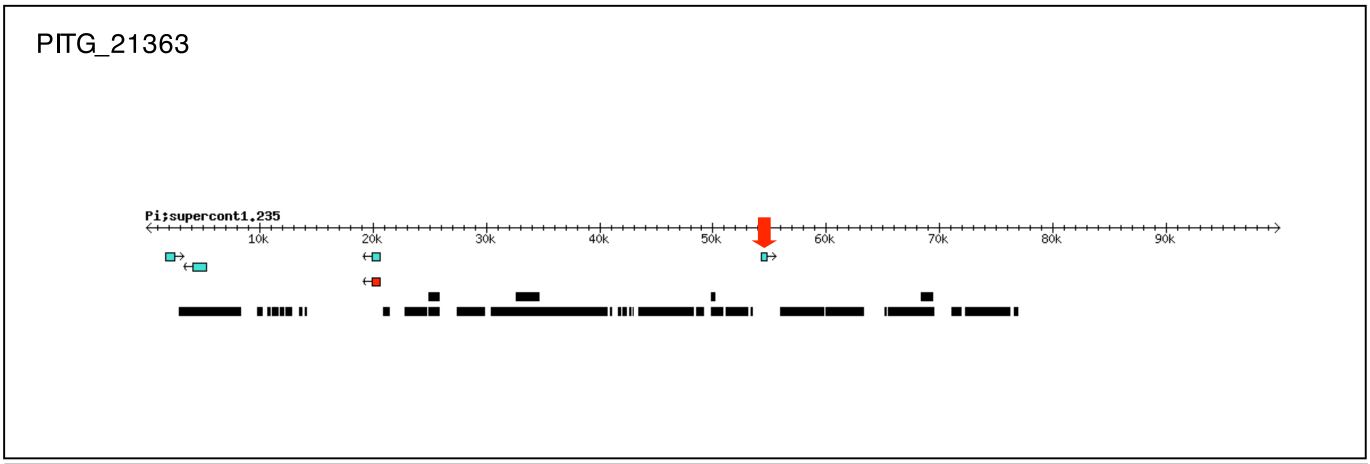

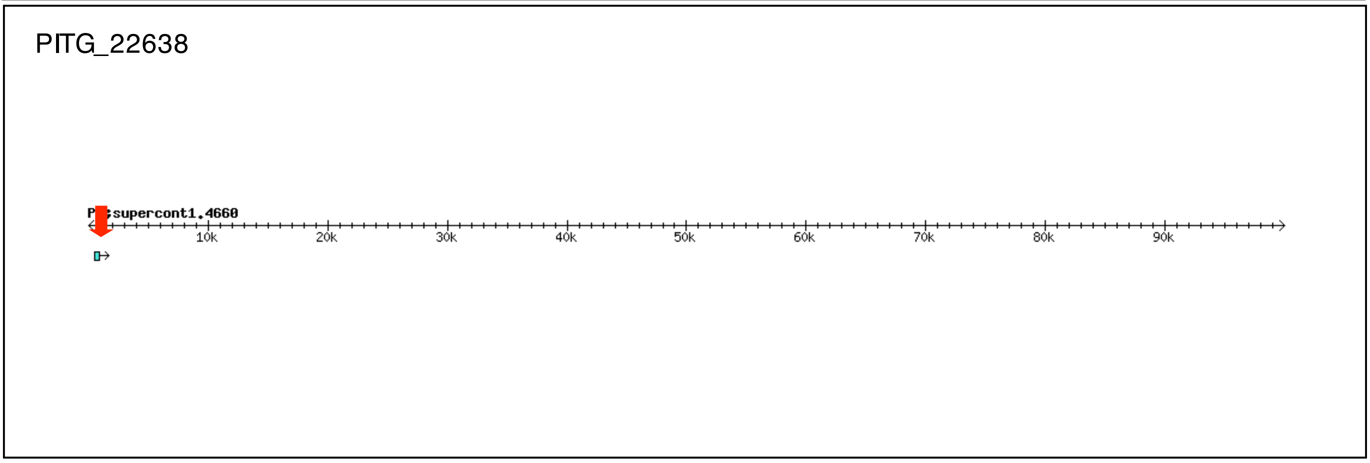

Figure S3 continued

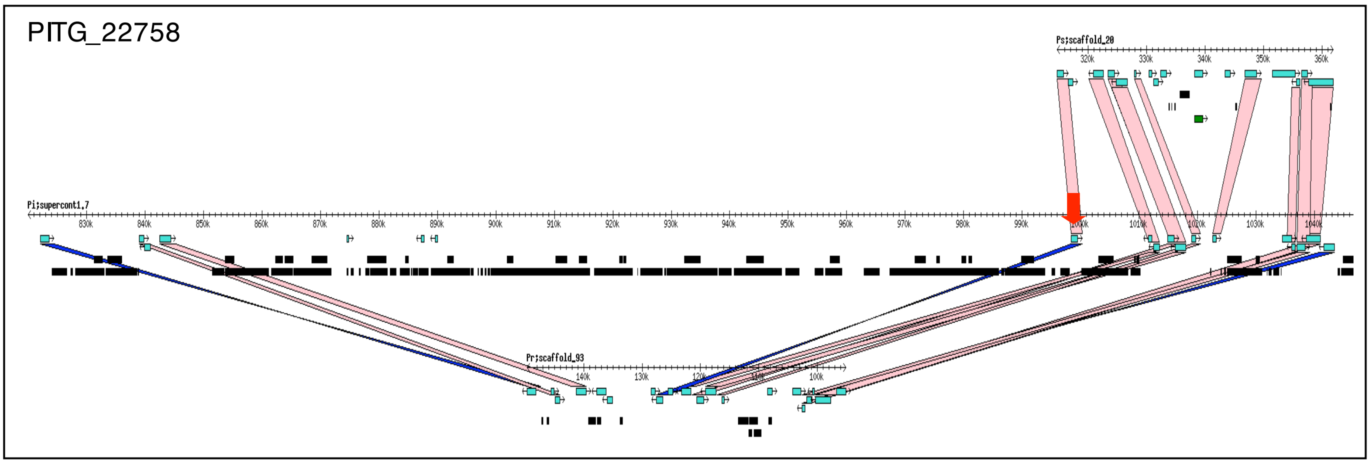

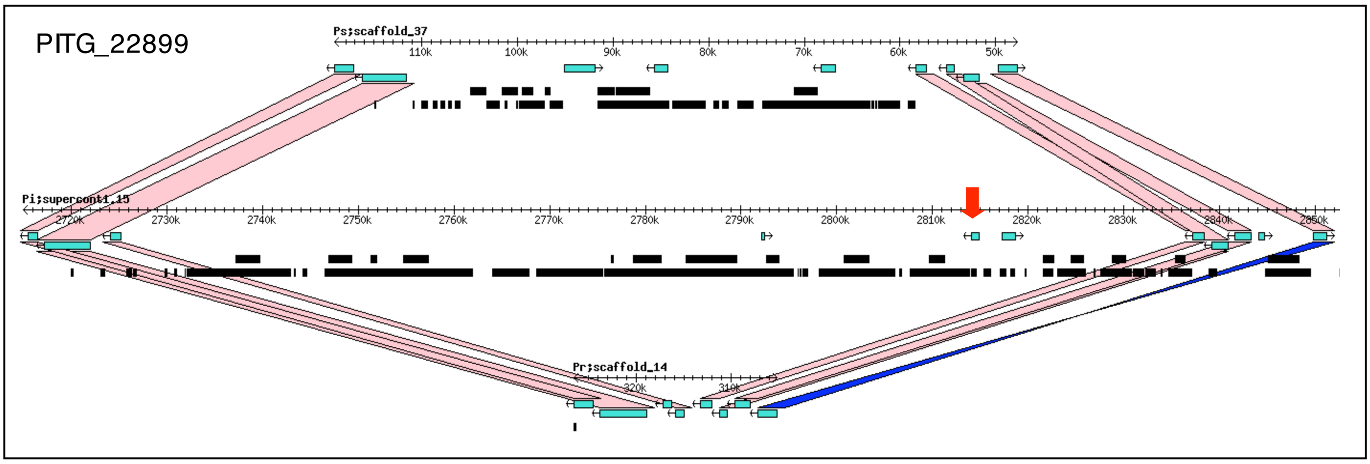

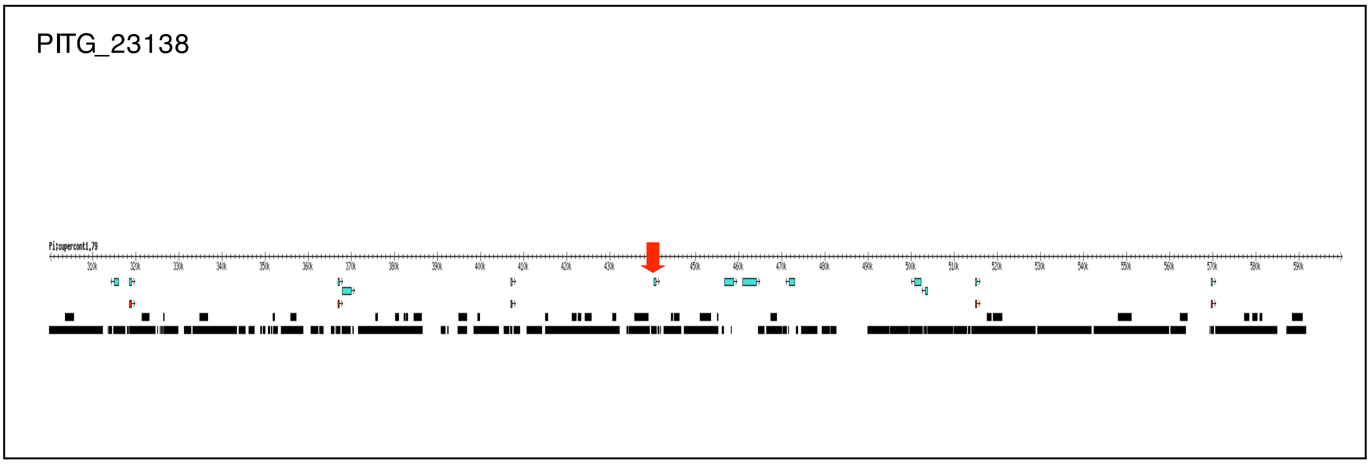

Figure S3 continued
