# Supplementary material for: Analyses of genome architecture and gene expression reveal novel candidate virulence factors in the secretome of Phytophthora infestans
Source: BMC Genomics. 2010 Nov 16;11:637. doi: 10.1186/1471-2164-11-637 (PMC3091767; doi:10.1186/1471-2164-11-637)
Supplement: Additional file 8 — Global analysis of P. infestans Small Cysteine-rich proteins. A) Distribution of P. infestans proteins according to their length and cysteine content. B) Position of PITG_04202 and other P. infestans secreted SCRs on the FIR heat map. C) Frequency of SCR sequences as a % of P. infestans whole proteome, secretome and non-secreted proteins; frequency of SCR genes induced as a % of SCR genes in P. infestans whole genome, secretome and non-secretome genes. [file 1471-2164-11-637-S8.PDF]

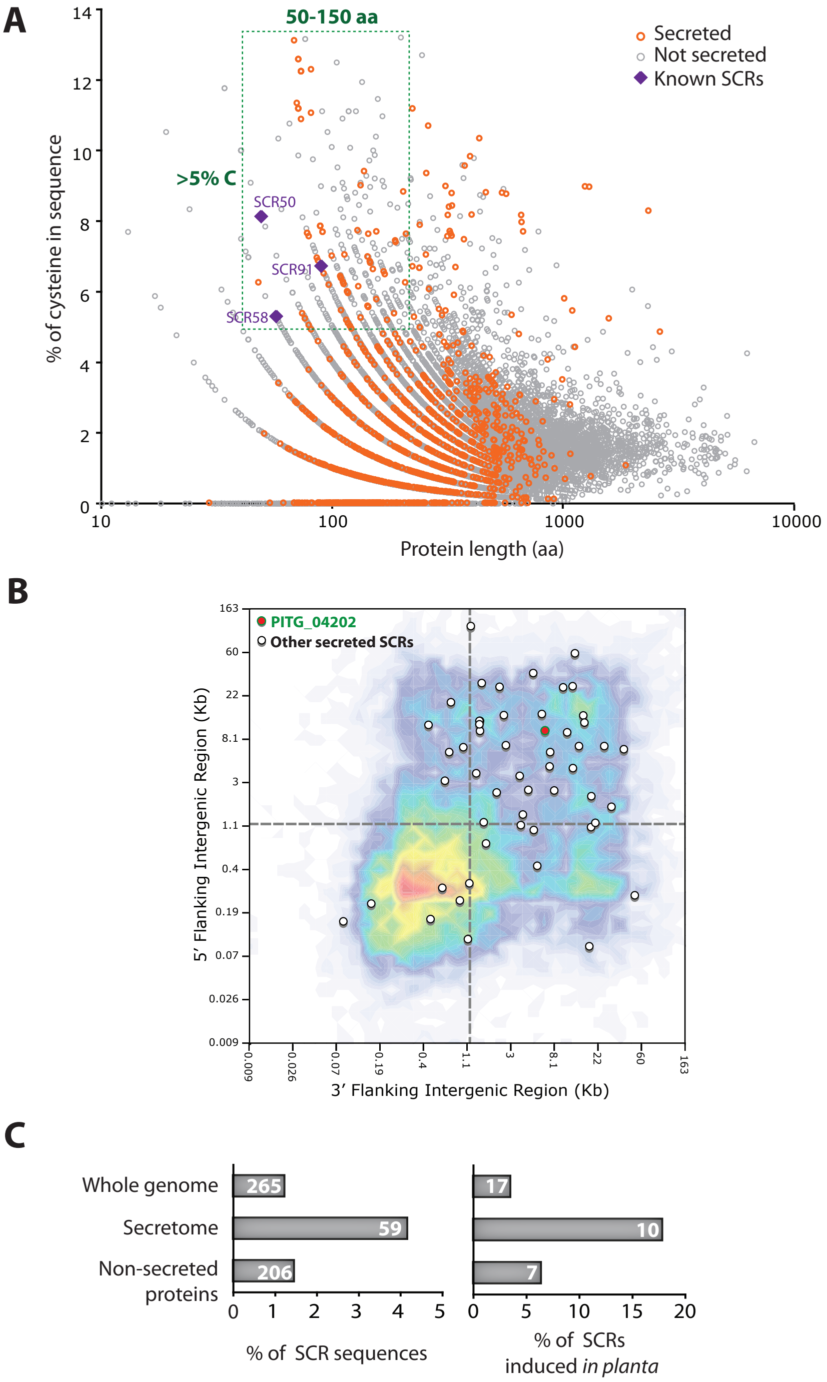

Additional file 8. Global analysis of *P. infestans* Small Cysteine-rich proteins

A) Distribution of *P. infestans* proteins according to their length and cysteine content.

B) Position of PITG_04202 and other *P. infestans* secreted SCRs on the FIR heat map (Figure 2B).

C) Frequency of SCR sequences as a % of *P. infestans* whole proteome, secretome and non-secreted proteins (left). Frequency of SCR genes induced as a % of SCR genes in *P. infestans* whole genome, secretome and non-secretome genes (right).
